# Panchromatic Ternary Polymer Dots Involving Sub-Picosecond Energy and Charge Transfer for Efficient and Stable Photocatalytic Hydrogen Evolution

**DOI:** 10.1021/jacs.0c12654

**Published:** 2021-02-04

**Authors:** Aijie Liu, Lars Gedda, Martin Axelsson, Mariia Pavliuk, Katarina Edwards, Leif Hammarström, Haining Tian

**Affiliations:** † Department of Chemistry-Ångström Lab., Uppsala University, Box 523, SE 751 20, Uppsala, Sweden

## Abstract

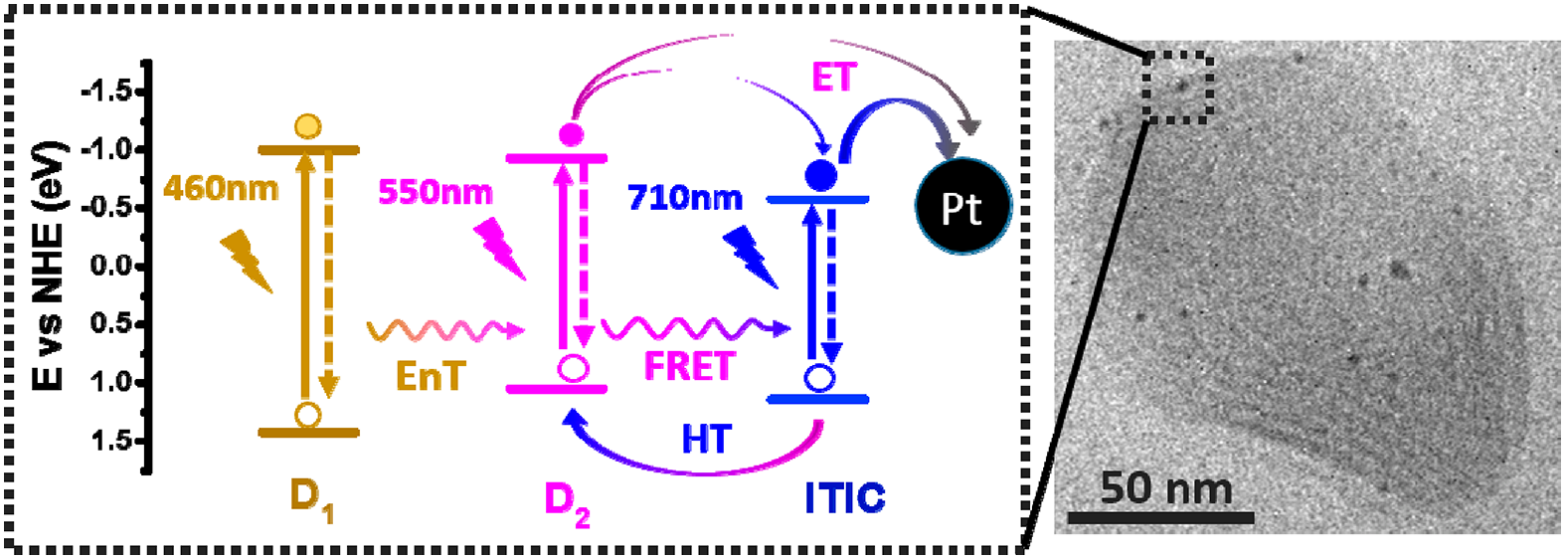

Panchromatic ternary polymer dots (Pdots) consisting of two conjugated polymers (PFBT and PFODTBT) based on fluorene and benzothiadiazole groups, and one small molecular acceptor (ITIC) have been prepared and assessed for photocatalytic hydrogen production with the assistance of a Pt cocatalyst. Femtosecond transient absorption spectroscopic studies of the ternary Pdots have revealed both energy and charge transfer processes that occur on the time scale of sub-picosecond between the different components. They result in photogenerated electrons being located mainly at ITIC, which acts as both electron and energy acceptor. Results from cryo-transmission electron microscopy suggest that ITIC forms crystalline phases in the ternary Pdots, facilitating electron transfer from ITIC to the Pt cocatalyst and promoting the final photocatalytic reaction yield. Enhanced light absorption, efficient charge separation, and the ideal morphology of the ternary Pdots have rendered an external quantum efficiency up to 7% at 600 nm. Moreover, the system has shown a high stability over 120 h without obvious degradation of the photocatalysts.

## Introduction

Global environment issues and increasing energy demands have led to an urgent requirement for clean and renewable energy to replace fossil fuel. Mimicking natural photosynthesis, the abundant solar energy can be converted and stored as chemical energy, such as solar hydrogen (H_2_). Molecular hydrogen produced directly from water has been recognized as one of the most promising green energy carriers, with high gravimetric energy density and no CO_2_ emission upon utilization.^1,2^ Besides photovoltaic-assisted electrolysis (PV-E) and photoelectrochemical (PEC) methods,^3^ photocatalysis from nanoparticles is also a promising approach for hydrogen generation from water.^4^ Since Fujishima and Honda first reported water splitting using TiO_2_ in the early 1970s,^5^ many robust and efficient inorganic photocatalysts have been reported.^6^ More recently, organic photocatalysts have been attracting attention as alternative materials, owing to their low cost, abundant resources, low toxicity, and facile tunable band gaps.^7,8^

Metal-free graphitic carbon nitride (g-C_3_N_4_) conjugated polymers are the most popular organic photocatalysts for hydrogen evolution with high photothermal stability.^9^ Results for newly developed covalent organic frameworks (COFs) with relatively high crystallinity have suggested high photoactive charge transport to the photoactive surface.^10^ However, most of the studies have estimated that the exciton diffusion path in organic semiconductors is limited to the range of 5–10 nm,^11,12^ implying that a small size of heterogeneous photocatalysts is preferred in order to decrease the required diffusion length and have sufficient charge separation at the interface of photocatalysts for the photocatalytic reactions. Recently, reports have shown that ultrathin g-C_3_N_4_ nanolayers are able to greatly enhance photocatalytic hydrogen evolution.^13,14^ Porous conjugated polymers as photocatalysts have been rapidly developed in recent years, and the structures of the polymer have been proven to be one of the key factors for obtaining good performance.^12−25^ Making the linear conjugated polymer particles as small as possible can shorten the required distance for exciton diffusion to suppress the unwanted annihilation of excitons as well as increase the catalytic surface area, therefore enhancing the photocatalytic performance. The nanoprecipitation method has been successfully used to make polymers into nanoparticles with sizes less than 100 nm.^26^ Amphiphilic polymers or surfactants are commonly used in this method to stabilize the polymer nanoparticles in water.^27^ Such prepared polymer nanoparticles are also named polymer dots (Pdots) and can have proton channels that are particularly beneficial for proton reduction.^28^ Therefore, Pdots have been successfully used as photocatalysts and have shown excellent performance for photocatalytic hydrogen evolution.^29−37^ Heterojunctions with donor and acceptor blending are another strategy to enhance charge separation, which has been widely studied in organic photovoltaics (OPVs).^38,39^ However, to date, only few studies on heterojunction Pdots have been reported for hydrogen production,^32,40,41^ which leaves room for exploration. Directions of interest include improving the stability for long-term use,^42^ understanding the photocatalytic mechanism of heterojunction Pdots, and increasing the light absorption for more efficient sunlight conversion. Moreover, to date, there is no energy transfer process introduced and investigated in the heterojunction Pdots for photocatalytic reactions. In this work, panchromatic ternary Pdots consisting of two organic polymers as energy and electron donors and a small molecule as energy/electron acceptor have been prepared and applied for photocatalytic hydrogen evolution with the assistance of a Pt cocatalyst, and have shown outstanding performance and stability. The energy and charge transfer pathways in the ternary Pdots have been investigated in detail.

## Experimental Section

### Materials

Semiconducting polymer poly[(9,9-dioctylfluorenyl-2,7-diyl)-*co*-(1,4-benzo-{2,1′,3}-thiadiazole)] (F8BT, also named as PFBT, D_1_, *M*_w_ 376 200) and n-type nonfullerene acceptor 3,9-bis(2-methylene-(3-(1,1-dicyanomethylene)indanone))-5,5,11,11-tetrakis(4-hexylphenyl)dithieno[2,3-d:2′,3′-d′]-*s*-indaceno[1,2-b:5,6-b′]dithiophene (ITIC) were purchased from Ossila, UK. PFODTBT polymer (D_2_, *M*_w_ 50–80 kDa) was purchased from Solaris Chem. The copolymer, polystyrene grafted with ethylene oxide and carboxyl groups (PS–PEG–COOH, backbone chain *M*_w_ 8500, graft chain *M*_w_ 4600, total chain *M*_w_ 36 500), was purchased from Polymer Source Inc., Canada. All other chemical reagents were purchased from Sigma-Aldrich and used as received unless indicated otherwise. All experiments and measurements were carried out at room temperature unless indicated otherwise.

### Preparation of Pdots

Pdots in aqueous solutions were prepared using a modified method according to the literature.^37,40^ In brief, PFODTBT was dissolved in tetrahydrofuran (THF) at a concentration of 100 μg mL^–1^, and PS–PEG–COOH, PFBT, and ITIC in THF at a concentration of 1.0 mg mL^–1^. PFODTBT, PFBT, and ITIC solutions were mixed in the ratio of the desired nanoparticle composition, and a PS–PEG–COOH solution was then added. In all nanoparticle precursor solutions, the weight ratio between PFODTBT and PFBT was kept at 3:2 wt/wt and PFODTBT:PS–PEG-COOH was kept at 1:3 wt/wt. In detail, 200 μL of PFBT, 3 mL of PFODTBT, 900 μL of PS-PEG-COOH and various amount of ITIC were mixed first and then sonicated for 2 min. The above mixture was then added into 8 ml deionized water (pre-heated at 85 °C), and kept under 85 °C for 45 min in order to completely remove THF. Pure ITIC dots was prepared by mixing 1 mL of ITIC with 1 mL of PS-PEG-COOH solutions, the rest procedures are same as binary/ternary Pdots. All samples were filtered through 0.45 μm syringe filter before further use. Final composition as well as concentration of Pdots solution was determined by follow: 100 μL of sample is freeze-dried by liquid nitrogen and dissolved in a certain amount of THF; then the solution was measured by UV-vis to determine the final weight of Pdots in the solution. The concentration of all Pdots solution for photocatalysis were eventually adjusted to 41 μg.mL^-1^ (without count of PS-PEG-COOH).

### Cryo-Transmission Electron Microscopy (Cryo-TEM)

Samples were analyzed by cryo-TEM as described earlier.^43^ Samples were equilibrated at 25 °C and high relative humidity within a climate chamber. A small drop of each sample was deposited on a carbon-sputtered copper grid covered with perforated polymer film. Excess liquid was thereafter removed by blotting with a filter paper, leaving a thin film of the solution on the grid. The sample was vitrified in liquid ethane and transferred to the microscope, continuously kept below −160 °C and protected against atmospheric conditions. Analyses were performed with a Zeiss Libra 120 transmission electron microscope (Carl Zeiss AG, Oberkochen, Germany) operating at 80 kV and in zero-loss bright-field mode. Digital images were recorded under low-dose conditions with a BioVision Pro-SM Slow Scan CCD camera (Proscan Elektronische Systeme GmbH, Scheuring, Germany).

### Dynamic Light Scattering (DLS) Measurements

The hydrodynamic diameter of samples was measured by a Zetasizer Nano-S from Malvern Instruments Nordic AB. Average data were obtained from at least five runs of measurements.

### Steady-State Absorption and Fluorescence Measurements

Steady-state UV–vis measurements were analyzed by using a PerkinElmer Lambda 750 UV–vis spectrophotometer. Steady-state fluorescence spectra were analyzed by using a Fluorolog 3-222 emission spectrophotometer (Horiba Jobin-Yvon) together with the FluorEssence software.

### Transient Absorption Spectroscopy (TAS)

The 1 mJ, 45 fs output of a 3 kHz Ti:sapphire amplifier (Libra coherent) was split into two separate commercial optical parametric amplifiers (TOPAS-C, Light Conversion), which generate the visible pump 460, 550, and 710 nm. Prior to reaching the sample, the probe beam was split into equal intensity probe and reference beams using a wedged ZnSe window. Only the probe beam that interacts with the photoexcited volume of the sample hits the detector. All beams are focused with a single *f* = 10 cm off-axis parabolic mirror to a 300 μm spot size in the sample. Both pump and probe lights are redirected to the Newport MS260i spectrograph with interchangeable gratings. The fundamental laser (probe, 795 nm) passes through the delay stage (8.5 ns and 1–2 fs step size) and is focused in a CaF_2_ optical window in order to generate UV–NIR light. The pump laser power was always kept at around 80 μW with less than 1% standard deviation (or as otherwise noted under figures), the pump beam profile is assumed to have a Gaussian distribution, the full width at half-maximum (fwhm) is around 300 μm, and the time resolution, i.e., the instrument response function, is ca. 180–200 fs. *f* = 1500 Hz. Pump scattering was autocorrected by TAS software during the measurements. The kinetic traces were fitted with a sum of convoluted exponentials:
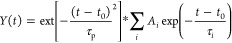
1where  and IRF is the width of the instrument response function (full width at half-maximum), *t*_0_ is the time zero, *A*_*i*_ and *τ*_*i*_ are amplitude and decay times, respectively, and * is the convolution operator.

### Spectroelectrochemistry

Both polymers were measured by coating polymers on fluorine-doped tin oxide glass (FTO) as working electrodes, were performed in acetonitrile (Sigma-Aldrich, anhydrous, 99.8%) with 0.1 M tetrabutylammonium hexafluorophophate (Sigma-Aldrich, electrochemical grade, dried at 80 °C in a vacuum), and purged with solvent-saturated nitrogen. Cyclic voltammograms were recorded in a standard three-electrode cell using an Autolab potentiostat (PGSTAT302) controlled with GPES software. The reference electrode was a nonaqueous Ag/Ag^+^ electrode, with a platinum (Pt) wire as counter electrode. UV–vis spectroelectrochemistry was performed with a glass cuvette with a 1 cm pass-length in a diode array spectrophotometer (Agilent 8453). Spectra were recorded with the electrode and with the same reference electrode used for voltammetry. Spectra were recorded in the case of controlled potential electrolysis using an Autolab PGSTAT100 potential station. For ITIC acceptor, dried THF was used as solvent, and a Pt wire flag was used as the working electrode; the rest are the same as the measuring polymers.

### Photocatalytic Hydrogen Generation

The photocatalytic hydrogen evolution was performed in 9 mL gastight vials. A 1.5 mL amount of Pdots (62 μg) with various ITIC weight ratios was first mixed with a specific amount of aqueous potassium hexachloroplatinate solution containing 4 μg Pt and purged with argon (Ar) for 20 min; then, 0.5 mL of prepurged ascorbic acid aqueous solution (0.8 M, pH 4 adjusted by 2 M KOH) was added into the above solution. The mixture was purged with Ar for another 30 min in order to completely remove oxygen. An LED PAR38 lamp (17W, 5000K, Zenaro Lighting GmbH, λ > 420 nm) was used as the light source. The light intensity illuminated on the active area of the sample was 50 mW cm^–2^, which was measured by a pyranometer (CM11, Kipp&Zonen, Delft/Holland). The LED light source basically has similar intensity to standard 1 sun condition between 420 and 750 nm. The generated hydrogen was quantified by an HPR-20 benchtop gas analysis system (HIDEN Analytical) using Ar as carrier gas.

### External Quantum Efficiency

External quantum efficiency (EQE) was tested by using 2 ml ternary Pdots with 55 wt % ITIC (0.2 M ascorbic acid, pH 4, 6 wt % of Pt) in a 3.5 mL airtight quartz cuvette (pass-length 1 cm). The solution is illuminated by 300 W Xe lamp (AULTT CEL-HXF300/CEL-HXUV300) as light source equipped an AM1.5 filter and different band pass filters (CEAULIGHT, 450, 500, 550, 600, 650, 700 and 765 nm) were used to select particular wavelength. The hydrogen was measured by an HPR-20 benchtop gas analysis system (HIDEN Analytical) using Ar as carrier gas. The EQE was calculated using the following equations 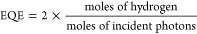

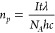
where *n_p_* represents the moles of incident photons, *I* is the radiant power, λ is the light wavelength, *t* is the irradiation time (excluding the induction time), *h* is the Planck constant, *N_A_* is the Avogadro constant, *c* is the speed of light.

### Powder X-ray Diffraction (PXRD)

PXRD patterns were collected at ambient temperature using a Simons D5000 diffractometer (Cu Kα, λ = 0.154 18 nm) at 45 kV and 40 mA, using a step size of 0.02° and a scan speed of 2 s per step in the range 2–50°. The diffractometer was equipped with parallel beam optics (mirror + mirror) for grazing-incidence XRD measurements.

## Results and Discussion

**Figure 1:**
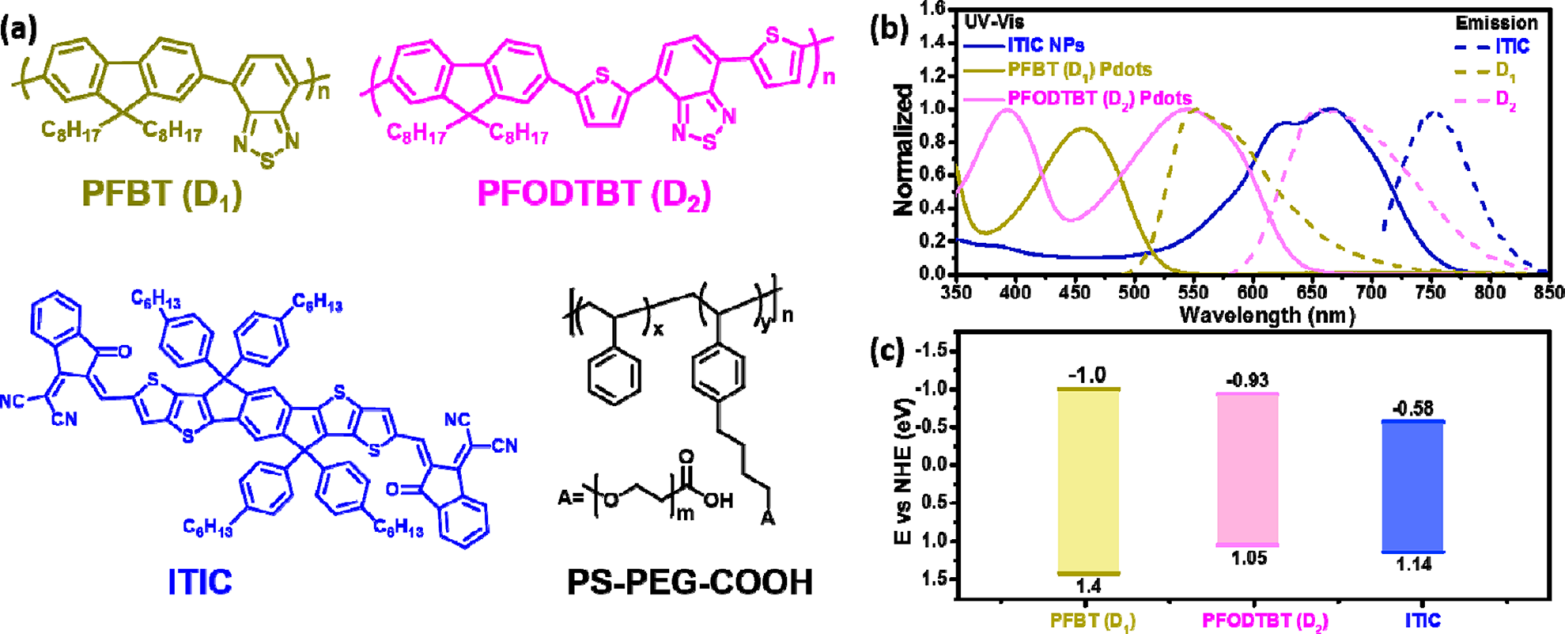
(a) Molecular structures of PFBT (D_1_), PFODTBT (D_2_), ITIC, and amphiphilic polymer PS–PEG–COOH. (b) Steady-state absorption (solid lines) and fluorescence spectrum (dashed lines) of D_1_, D_2_, and ITIC. (c) Energy diagram of D_1_, D_2_, and ITIC.

The structures of all components involved in the panchromatic ternary Pdots are shown in Figure 1a. The amphiphilic polymer PS–PEG–COOH, which itself shows no light absorption or photochemical activity within the visible light region, was used to stabilize the Pdots in aqueous solution. Two polymers, PFBT (D_1_) and PFODTBT (D_2_), were used as energy and electron donors (D), together with the molecular acceptor ITIC. From Figure 1b, one can see that D_1_, D_2_, and ITIC have excellent complementary absorption spectra up to 770 nm (absorption spectra of D_1_, D_2_ and ITIC in THF are shown in Figure S1). Interestingly, D_1_ has strong fluorescence emission between 500 and 700 nm that largely overlaps with the absorption spectra of both D_2_ and ITIC. This large spectral overlap is normally essential for efficient Förster resonance energy transfer (FRET), if the distance between corresponding components is similar to, or smaller than, the characteristic Förster distance of the donor–acceptor pair.^44^ From the energy levels shown in Figure 1c, there are two types of heterojunctions that are likely to form in the ternary Pdots: a straddling gap (type I, between D_1_ and D_2_ and between D_1_ and ITIC) that allows energy transfer (EnT) and a staggered gap (type II, between D_2_ and ITIC) that could render charge transfer reactions, including both and hole transfer (HT), at the interfaces upon different light excitations. It is scientifically interesting to investigate the potential EnT and charge transfer pathways in the ternary Pdots. Moreover, energy and charge transfer in ternary Pdots should enhance light utilization and prolong the lifetime of charge carriers, which is therefore beneficial for improving photocatalytic reaction performance such as hydrogen production in this work.

### Ternary Pdot Preparation and Characterization

**Figure 2:**
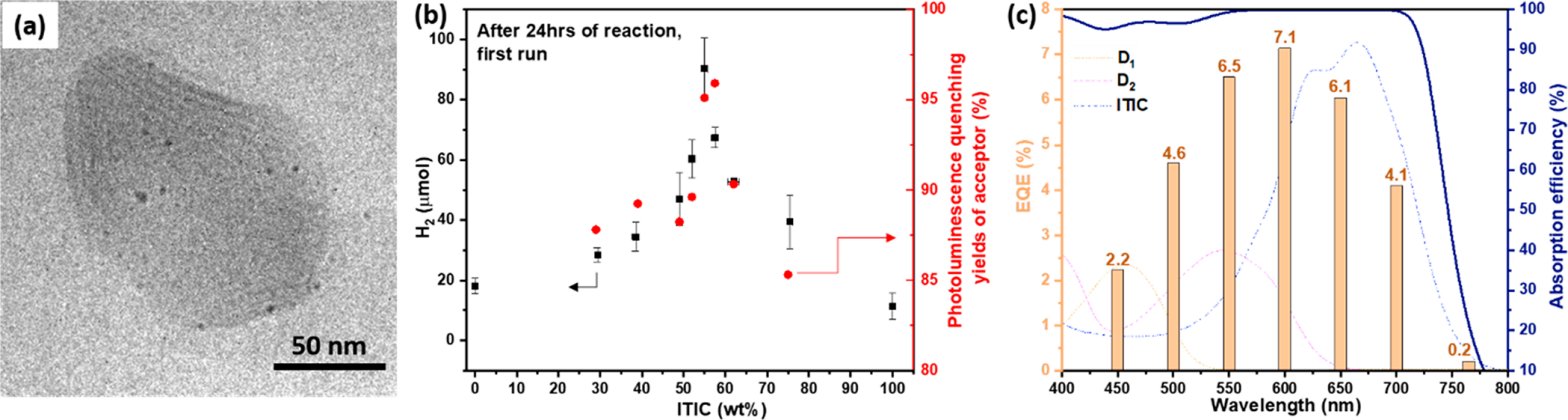
(a) Cryo-TEM micrograph of a ternary Pdot prepared in the presence of 55 wt % ITIC. (b) Average amount of H_2_ produced over 24 h of different samples and photoluminescence quenching yields of ITIC in various ternary Pdots as a function of ITIC mass ratio. (c) EQEs of the optimal ternary Pdots at absorption wavelengths of 450, 500, 550, 600, 650, 700, and 765 nm. Dashed lines presented at the background of (c) are absorptions for individual D_1_, D_2_, and ITIC Pdots.

The ternary Pdots were prepared by a modified nanoprecipitation method reported in previous publications.^26,45^ A series of Pdots with varying amounts of ITIC and with an average hydrodynamic diameter of 90 nm were prepared (Figure S2). Cryo-TEM was used to study the morphology of Pdots. The ternary Pdots display an irregular shape and a possibly layered morphology (Figure S3). This is also seen for ternary Pdots with an in situ photodeposited Pt cocatalyst, where the Pt nanoparticles (NPs) are homogeneously decorated around Pdots (Figure 2a). The crystalline nature of the particles is most likely due to the presence of ITIC, since this small molecule tends to form crystalline structures,^46,47^ and it is further supported by XRD measurements (Figure S4). In contrast, only spherical and amorphous particles were found for D_1_/D_2_ binary Pdots (Figure S5).

### Photocatalytic H_2_ Production

A series of ternary Pdots containing varying amounts of ITIC were tested for photocatalytic hydrogen evolution reaction (HER) with 6 wt % Pt as cocatalyst, as shown in Figure S6. An ITIC content of 55 wt % was found to give optimal HER activity of ternary Pdots (Figure 2b), showing a maximum HER rate of 60.8 ± 6.7 mmol h^–1^ g^–1^ (organic photocatalyst) and 1.88 ± 0.21 μmol h^–1^ mL^–1^ (reaction solution). Interestingly, the HER rate of Pdots with various amounts of ITIC shows a similar trend to that for the ITIC fluorescence quenching yield (red circle in Figure 2b). This result is consistent with recently published literature,^40^ in which the most efficient exciton dissociation was observed at a blend ratio giving the highest HER, indicating that efficient charge generation is essential in photocatalytic reactions. In addition, the morphology of Pdots with ITIC can strongly reduce the distance between ITIC and Pt NPs, which may facilitate charge transfer from reduced ITIC (ITIC^•–^) to the Pt cocatalyst for hydrogen evolution. The ternary Pdots with 55 wt % ITIC were used to measure EQEs of the photocatalytic reaction. As shown in Figure 2c, a relatively high EQE was obtained within a wavelength range from 450 to 700 nm, which matches the maximum sunlight irradiation flux region. The maximum EQE obtained by the ternary Pdots is 7.1% at 600 nm (Figure 2c). In order to get insights into the mechanism of energy and charge transfer in the system, both steady-state and transient spectroscopic studies of the system were carried out.

### Steady-State UV–Vis and Fluorescence Study of Ternary Pdots

**Figure 3:**
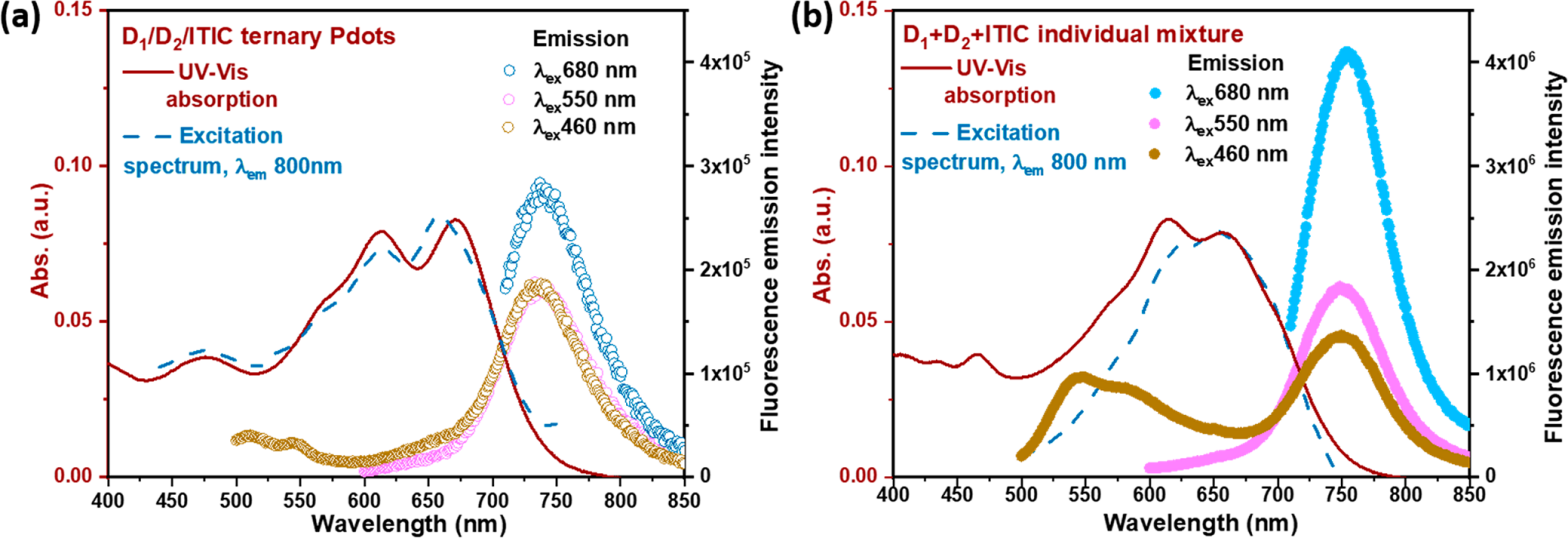
Steady-state UV–vis and fluorescence study of (a) ternary Pdots. The absorption spectrum is shown as a solid line (dark red). Fluorescence emission was taken with excitation of 460 nm (yellow empty circle), 550 nm (purple empty circle), and 680 (sky blue empty circle) and the fluorescence excitation spectrum was taken with emission at 800 nm (dashed line). (b) Individual mixtures of D_1_ Pdots, D_2_ Pdots, and ITIC NPs with equal concentration to ternary Pdots.

Figure 3a shows the steady-state absorption and fluorescence spectra of the optimized ternary Pdots containing 55 wt % ITIC for the above photocatalytic reaction. A fluorescence study of the ternary Pdots was carried out with different excitation wavelengths of 460, 550, and 680 nm, which are the maximum absorption peaks for D_1_, D_2_, and ITIC, respectively (note that 460 nm is also able to excite D_2_, and both 460 and 550 nm are also able to excite ITIC). For comparison, fluorescence was also measured from single-component Pdots mixed at equal concentrations, as shown in Figure 3b. We notice that under all excitation wavelengths, the fluorescence emission (FE) intensities of D_1_ and ITIC in ternary Pdots were both strongly quenched, by at least 1 order of magnitude (Figure 3a), compared to the FE intensities of mixed individual Pdots (Figure 3b). Note that the D_2_ fluorescence is very weak also in single-component Pdots. For ternary Pdots, the following quenching mechanisms are energetically possible, based on the HOMO and LUMO levels of the individual components. Excitation EnT (Förster and/or Dexter) can happen in an excitation sequence excited D_1_ (D_1_*) → excited D_2_ (D_2_*) → excited ITIC (ITIC*), as well as directly from D_1_* → ITIC*. In addition, several excited-state charge transfer processes are possible. D_1_* can be quenched by either electron transfer (ET) or HT by both D_2_ and ITIC. For D_2_*, charge transfer quenching can only occur by ET to ITIC, and for ITIC*, HT to D_2_ is the only CT possibility; both processes lead to the same oxidized (D_2_^+^) and ITIC^•^^–^ products.

The fluorescence excitation spectra of the ternary Pdots provide strong evidence for efficient EnT. The excitation spectrum with emission at 800 nm (ITIC fluorescence) gives similar features to the absorption spectrum, as shown in Figure 3a (dashed line). This suggests efficient EnT from the other components to ITIC. In contrast, only the ITIC absorption feature was monitored in the excitation spectrum of the mixture of individual Pdots (Figure 3b, dashed line), indicating no EnT proceeded in the Pdots mixture, likely due to an unfavorable donor–acceptor distance. Under excitation at 680 nm (λ_ex_ = 680 nm is not able to excite both D_1_ and D_2_), 94% of ITIC FE intensity (Figure 3a) was quenched in the ternary system, in comparison with the mixed single-component Pdots system (Figure 3b). This is attributed to photoinduced HT from ITIC* to D_2_, which indicates a sufficient exciton separation at the interface of the D_2_/ITIC heterojunction.

**Figure 4:**
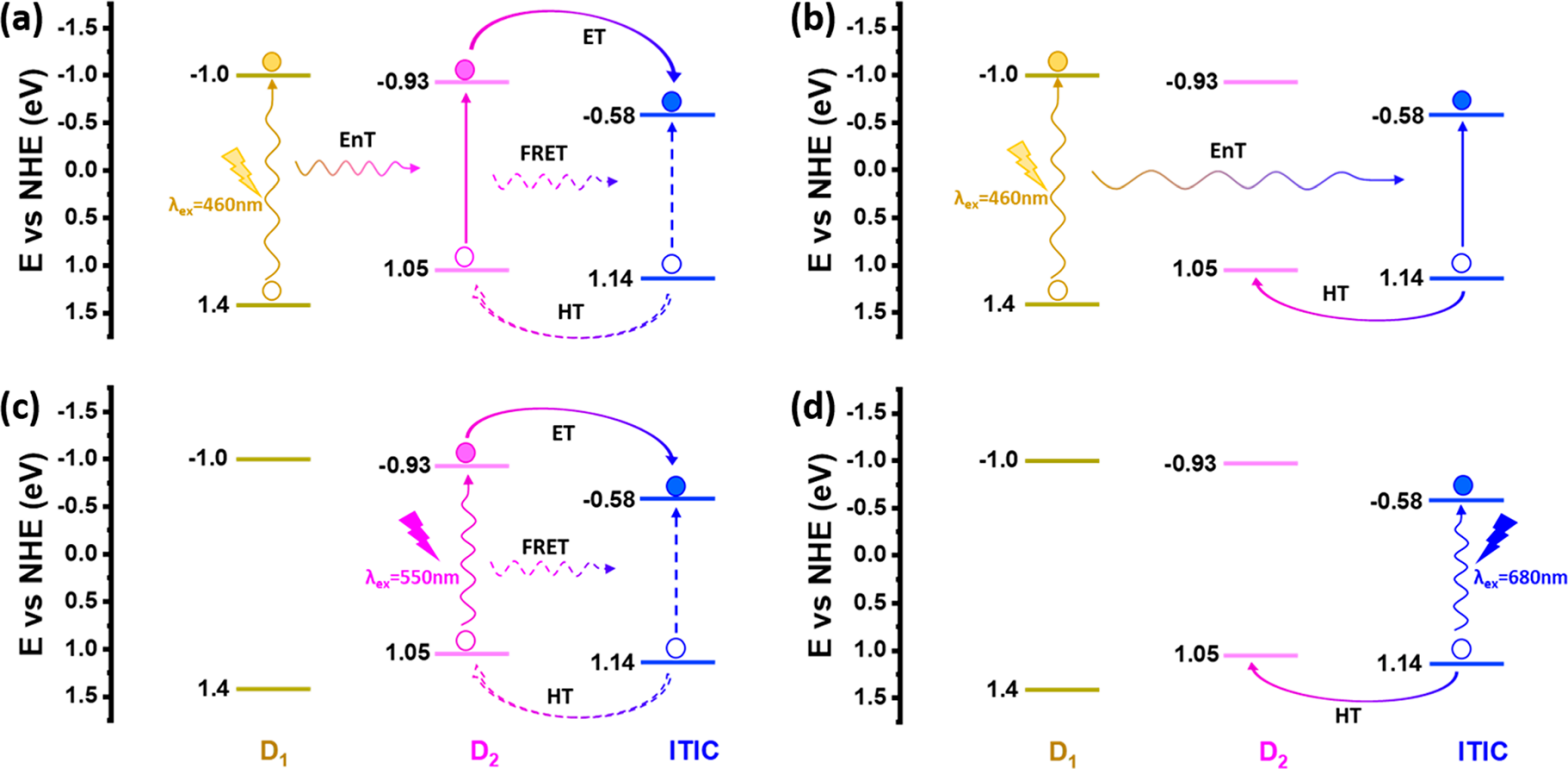
Proposed energy and charge transfer pathways between D_1_, D_2_, and ITIC in ternary Pdots under the excitation of 460 nm (a and b), 550 nm (c), and 680 nm (d). Dashed arrows in (a) and (c) present a parallel two-step pathway from D_2_ to ITIC: FRET from D_2_* to ITIC first, followed by HT from ITIC* to D_2_.

According to the above results, we can draw a primary framework of photophysical energy and charge transfer pathways between D_1_, D_2_, and ITIC in the ternary Pdots, as shown in Figure 4. Under excitation of 460 nm, the remaining possible pathways according to Figure 4a are as follows: first, EnT (might include both Dexter EnT and FRET) from D_1_* to D_2_; second, one-step ET from D_2_* to ITIC; and/or a two-step process involving FRET from D_2_* to ITIC, followed by HT from ITIC* to D_2_. In parallel, possible pathways according to Figure 4b are EnT from D_1_* to ITIC followed by HT from ITIC* to D_2_. With excitation of 550 nm shown in Figure 4c, again, both one-step ET from D_2_* to ITIC and the two-step process are possible. Under excitation of 680 nm, photoinduced HT is the only pathway that can happen from the ITIC* to D_2_ (Figure 4d). In order to further clarify the EnT and charge transfer pathways in the ternary Pdots and give a clear picture of mechanisms as well as their dynamics, we therefore prepared various binary Pdots of D_1_/ITIC, D_2_/ITIC, and D_1_/D_2_ to perform corresponding photophysical studies. The kinetics of the photophysical pathways was studied by TAS with pump wavelengths at the maximum absorption of individual components and a probe interval of 350–750 nm. Individual Pdots studies including both TAS and spectroelectrochemistry are presented in the Supporting Information, as shown in Figures S7 and S8, respectively.

### Energy Transfer

Both D_1_/ITIC and D_1_/D_2_ binary systems form straddling gap (type 1) heterojunctions;^48^ therefore, D_1_ here plays a role of light harvester in the wavelength range of 400–500 nm. Steady-state FE and excitation spectra of D_1_/ITIC and D_1_/D_2_ are shown in Figures S9 and S10, respectively. According to the steady-state absorption spectra, the photon absorption efficiency at 460 nm is 94% for D_1_ in D_1_/ITIC and 82% for D_1_ in D_1_/D_2_ binary Pdots, as shown in Figures S9a and S10a, respectively. The intensities of D_1_ FE with and without ITIC or D_2_ were compared, both in binary Pdots and in samples where single-component Pdots were mixed at equal concentrations (mixed individual Pdots). Note that the FE quenching of D_1_ was only observed in the binary Pdots, in both D_1_/ITIC and D_1_/D_2_ systems, with an accompanying increase in FE intensity of ITIC and D_2_, as shown in Figure S9b and S10b, respectively. No FE quenching of D_1_ was observed in the mixed individual Pdots (Figure S9b and S10b). The results indicate that EnT is the main quenching event of D_1_* in both D_1_/ITIC and D_1_/D_2_ binary Pdots due to the shorter distance between each component. This is further supported by excitation spectra under emission wavelengths of 820 and 780 nm, which showed similar features to the absorption spectra of D_1_/ITIC and D_1_/D_2_ binary Pdots, respectively (see Figure S9c and S10c).

**Figure 5:**
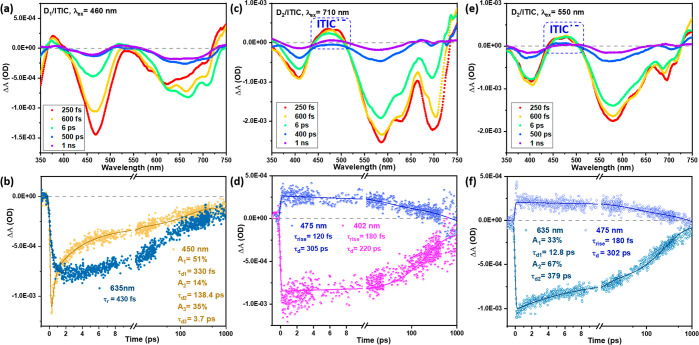
TA spectra of binary Pdots. (a) D_1_/ITIC under excitation 460 nm; (b) kinetics probed at 635 and 450 nm of (a); (c) D_2_/ITIC under excitation of 710 nm and (d) kinetics probed at 475 and 402 nm; (e) D_2_/ITIC under 550 nm excitation and (f) kinetics probed at 635 and 475 nm of (e).

The kinetics of EnT within D_1_/ITIC and D_1_/D_2_ binary Pdots were further studied by TAS with a pump wavelength of 460 nm and a probe interval of 350–750 nm. In this probe region, ground state bleach (GSB) features of D_1_ at 475 nm, ITIC at 635 and 700 nm (Figure 5a), and D_2_ at 575 nm (Figure S11a) were monitored. Neither clear oxidized nor reduced species signals were found in any systems when we compared with spectroelectrochemistry analysis (Figure S8). Thus, these results are consistent with the steady-state study showing that EnT is the main photoinduced event for both D_1_/ITIC and D_1_/D_2_ binary Pdots, with at least 85% (according to the degree of D_1_ FE quenching in binary Pdots) of the excitons generated by D_1_ being transferred in both binary systems. Due to low absorption of ITIC and D_2_ at the pump wavelength, direct excitation of ITIC and D_2_ is rather weak; therefore GSB of both ITIC and D_2_ mainly result from EnT of D_1_*. Both the recovery dynamics of D_1_ GSB and the formation dynamics of ITIC* and D_2_* reflect the EnT rate. All TAS kinetics were fitted by using a sum of exponential functions convoluted with the instrumental response function (see eq 1). The recovery of the D_1_ GSB was with lifetimes of 330 fs (51%) and 3.7 ps (35%) for D_1_/ITIC binary Pdots and 390 fs (91%) for D_1_/D_2_ binary Pdots. This was accompanied by ultrafast induction of GSB for either ITIC or D_2_, with similar rise times of 430 and 320 fs, respectively (Figure 5b and Figure S11b, respectively). This gives further evidence for EnT in these binary Pdots. The sub-picosecond EnT is presumably due to a sufficiently short distance between the components in the binary Pdots.

### Hole Transfer in D_2_/ITIC Pdots

In D_2_/ITIC Pdots, under excitation at 710 nm, where ITIC is selectively excited, photoinduced HT from ITIC* to D_2_ is then the only deactivation pathway in the D_2_/ITIC heterojunction. Interestingly, in addition to GSB of ITIC, an additional negative peak 400 nm and a shoulder at 525 nm, as well as positive absorption between 440 and 510 nm, all appeared immediately after the excitation (Figure 5c), with a rise time below our instrument response function (τ ≈ 200 fs) (Figure 5d). The negative absorption at 400 and 525 nm can be assigned to GSB of D_2_^+^ (see spectroelectrochemistry study in Figure S8). The positive absorption between 440 and 510 nm matches well with the absorption spectrum of ITIC^•–^ obtained from spectroelectrochemisty (Figure S8), considering that the net absorption change due to D_2_^+^ at 475 nm is negative (Figure S8) and the sum of photoinduced absorption of ITIC* and GSB of ITIC at 475 nm is close to zero (Figure S7). Ultrafast charge transfer (<100 fs) was also observed in conjugate polymer/fullerene binary nanoparticles,^49^ that were prepared by the nanoprecipitation method. Intrinsic HT on the sub-picosecond time scale (400 fs) under near-zero driving force (0.05 eV) was also observed elsewhere in polymer donor/nonfullerene acceptor blend thin films.^50,51^ Moreover, efficient charge transfer at low driving force and sub-picosecond long-range charge separation up to 5 nm within 40 fs has been observed and has been suggested to be facilitated by an intermolecular electric field that is the result of different electrostatic potentials between the donor and acceptor.^52,53^

### Electron Transfer versus Energy Transfer from D_2_ to ITIC

Subsequently, we investigate ET and EnT from D_2_ to ITIC by both steady-state fluorescence and TAS studies. Under excitation at 550 nm, 85% of photons are absorbed by D_2_ and 15% of photons are directly absorbed by ITIC, according to the absorption spectrum in Figure S12a. In addition to the possible ET from D_2_* to ITIC according to energy level shown in Figure 1, FRET from D_2_* to ITIC is also possible considering the large overlap between singlet exciton emission of D_2_ and absorption of ITIC. Under the excitation of 550 nm, FE intensities of both D_2_ and ITIC in binary D_2_/ITIC Pdots (Figure S12b) were highly quenched compared to FE in the individual Pdot mixture (D_2_ Pdots and ITIC NPs, Figure S12c). Quenching of both components can be a result of a two-step process of FRET from D_2_* to ITIC followed by HT from ITIC* to D_2_, possibly in parallel to one-step ET from D_2_* to ITIC. The potential FRET from D_2_* to ITIC is supported by the excitation spectrum shown in Figure S12b, which gives similar features to the absorption spectrum of D_2_/ITIC binary Pdots. As a comparison, the excitation spectrum of the mixed D_2_ Pdots and ITIC NPs shows a lack of a characteristic D_2_ peak at 550 nm (Figure S12c). In order to further clarify the potential pathway and their kinetics, a TAS experiment was performed. Under the pump of 550 nm, both positive and negative absorption reached the maximum within 250 fs (Figure 5e). The ITIC^•–^ (450 to 500 nm) appeared at a very early time after excitation, and fitting the rising edge region by a monoexponential function with convolution of the IRF gave a rise time of 180 fs (Figure 5f), similar to our IRF (τ ≈ 200 fs). This rise could suggest one-step ET from D_2_* to ITIC is probably the main event under excitation of 550 nm. High exciton delocalization in the conjugated polymer has been suggested to explain such a sub-picosecond ET process without the need of exciton diffusion.^54,55^ However, a two-step process, as suggested by the fluorescence excitation spectra, is also possible if the overall rate of FRET from D_2_* to ITIC and HT from ITIC* to D_2_ is higher than the time resolution of femtosecond spectroscopy.

### TAS Study of Ternary Pdots

**Figure 6:**
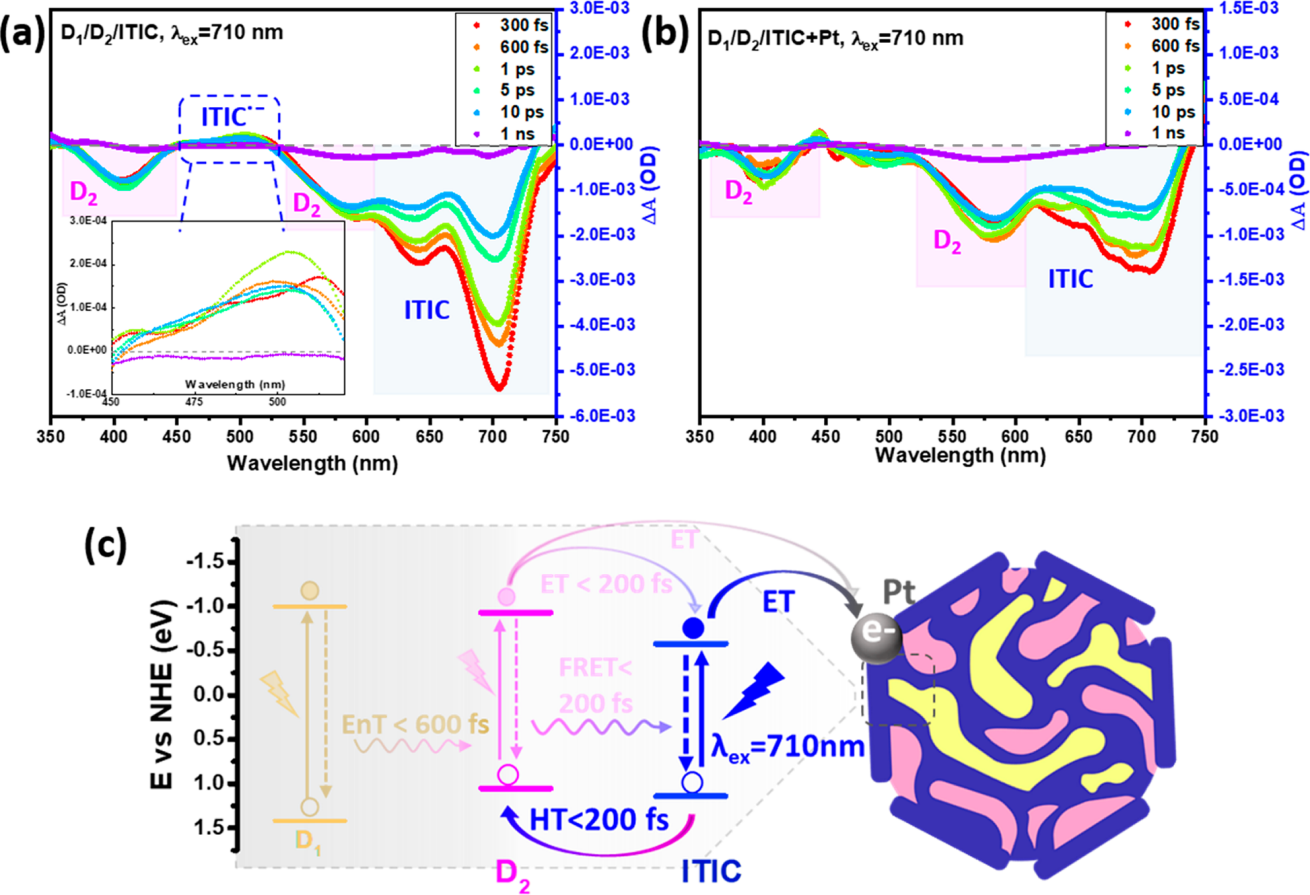
TA spectra of systems under excitation of 710 nm. (a) D_1_/D_2_/ITIC ternary Pdots and (b) D_1_/D_2_/ITIC ternary Pdots with in situ Pt deposited hybrids. (c) Scheme of photophysical pathways. The highlighted part indicates processes for (a) and (b), while pathways with a shaded background are presented in detail in the Supporting Information (Figure S18).

After understanding the photophysical pathways between D_1_, D_2_, and ITIC, we continued with a TAS study of ternary Pdots. For the convenience of the study, ternary Pdots with 29 wt % of ITIC were studied here, since this system gives similar absorption intensity at 460, 550, and 710 nm (Figure S13). Similar photophysical pathways to those described above were also found in ternary Pdots: ultrafast EnT from D_1_* to D_2_ and/or ITIC under excitation at 460 nm (Figure S14a); one step ET from D_2_* to ITIC and a two-step process involving FRET from D_2_* to ITIC followed by HT from ITIC* to D_2_ under excitation of 550 nm (Figure S14b); and HT from ITIC* to D_2_ under excitation of 710 nm (Figure 6a) were all observed. The influence of the Pt cocatalyst was also studied by comparing Pdots with and without in situ photodeposited Pt. It is possible to observe from the spectrum that when Pt cocatalyst is present, ITIC GSB intensities were significantly reduced at the early time under all excitation wavelengths. Here we take the excitation of 710 nm as an example, shown in Figure 6a and b. The TAS of the ternary system under 480 and 550 nm excitations are shown in Figure S14c,d. The observed intensity reduction can be explained by ultrafast ET from ITIC^•–^ to the Pt cocatalyst. In order to understand interactions between Pt and D_1_ as well as D_2_, kinetics studies of GSB recovery of D_1_ and D_2_ in systems with and without Pt were therefore compared. For D_1_ in ternary Pdots, similar kinetics of D_1_ GSB recovery in systems with and without Pt cocatalyst were obtained, which suggests that there is no (or only marginal) direct charge transfer between D_1_ and Pt on the time scale studied (ca. 5 ns, Figure S15). This is in accordance with the fact that D_1_ shows highly efficient EnT to D_2_ and ITIC, with at least 85% occurring in the time component of 600 fs or less (Figure S15). The same was observed for D_2_ in ternary Pdots excited at 710 nm (Figure S16a–c), indicating that there is no direct charge recombination between the oxidized D_2_ and the reduced Pt. However, a faster recombination in the system with Pt was observed with excitation at 460 nm (Figure S16d–f) and 550 nm (Figure S16g–i), which is evidence of direct reaction between D_2_ and Pt. This faster recombination is probably due to an additional ET process from D_2_* to Pt, which results in hole accumulation in D_2_, and this further leads to an accelerated recovery of D_2_ GSB. This assumption was supported by the results shown in Figure S17, where slower decay kinetics was obtained after adding the hole scavenger ascorbic acid. That both hole and electron accumulation may result in faster recombination has been reported by others.^56,57^ Another important point to note is that Pt cocatalyst as an additional electron acceptor allows greater spatial charge separation and prevents fast charge recombination between D_2_^+^ and ITIC^•–^. This is shown by the data in Figure 6b, with an increase in GSB intensity of D_2_ (525–610 nm) up to one picosecond, while this growth of D_2_ GSB was not observed in bare ternary Pdots (Figure 6a). Overall, the above results suggest that energy and charge transfer pathways between D_1_, D_2_, and ITIC in ternary Pdots basically follow the same framework as we suggested before. As shown in Figure 6c, ITIC plays a role as an electron and energy acceptor, resulting in a successful in situ photodeposition of Pt cocatalyst.^57,58^ Considering the principle of the photodeposition method and photophysical mechanism in this study, the results suggest that Pt NPs probably mainly deposited on/or close to the ITIC phase, which therefore promotes the photocatalytic reaction.

### Photocatalytic Stability of Ternary Pdots

**Figure 7:**
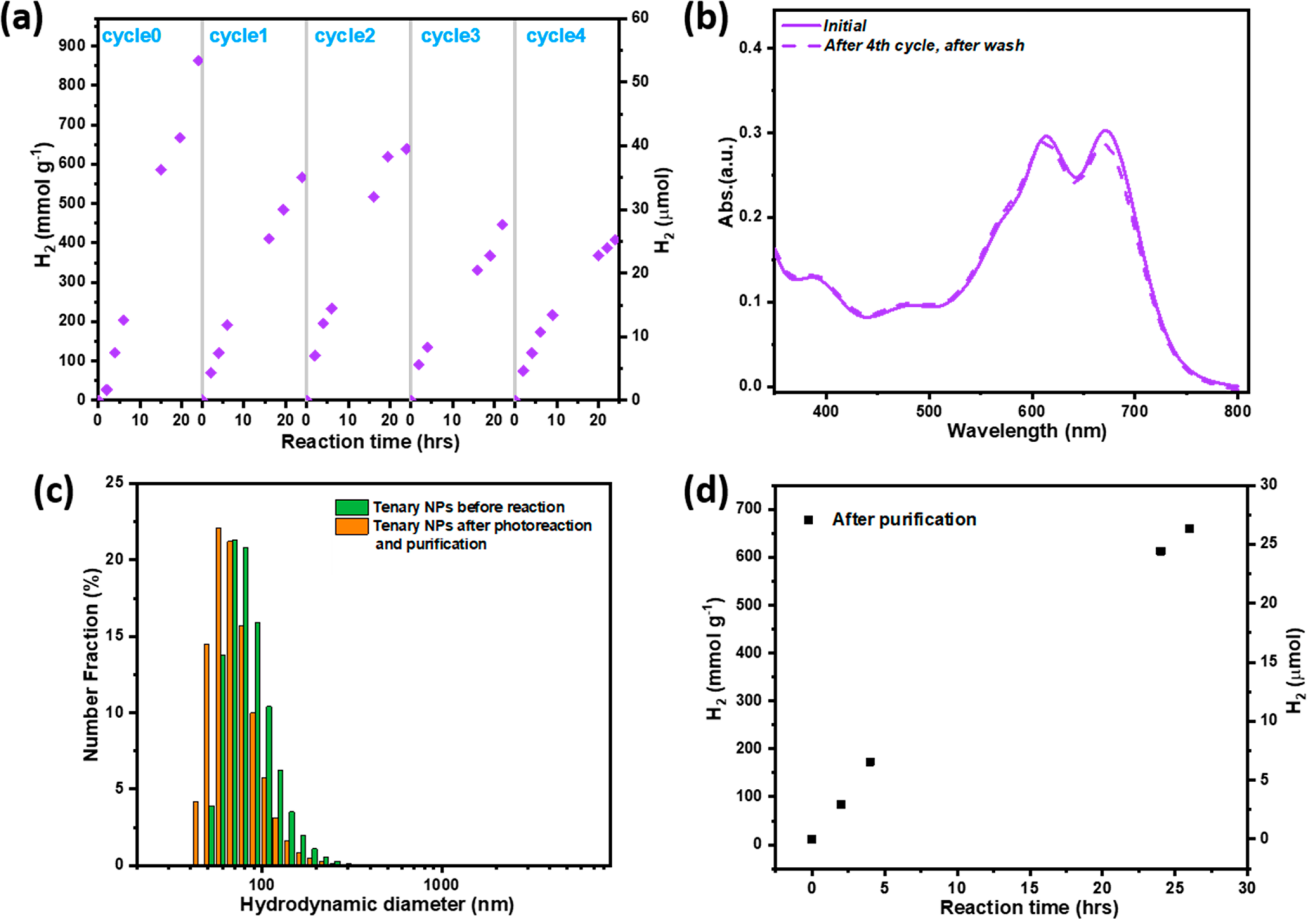
(a) Recycling experiment of hydrogen evolution. A 35 mg amount of ascorbic acid was added after cycle 1 and cycle 3, and the final pH was adjusted to pH 4 with 2 M KOH solution. (b) UV–vis analysis of ternary Pdots before reaction (solid line, diluted 15 times) and the 4th cycle of reaction and purification (dashed line, diluted 10 times). (c) DLS measurement of ternary Pdots before reaction and after reaction and purification. (d) Photocatalytic study of ternary Pdots. Samples were collected after the 4th cycle of reaction and purification. Reaction condition: a certain amount of ascorbic acid was added to the washed solution, 2 mL in total, 0.2 M ascorbic acid, pH 4. Note: final concentration of Pdots decreased after the washing steps; the final concentration is 60% of the initial concentration.

Finally, the photocatalytic stability of the ternary Pdots with 55 wt % of ITIC was evaluated. The reaction solution was purged with 99.99% argon (Ar) after every 24 h of reaction due to the detection limit of gas chromatography (GC). The amount of hydrogen produced along with reaction time and the stability test are shown in Figure 7a. The reactivity of the ternary Pdots decreased by about 35% after cycle 1 probably due to the lack of ascorbic acid. Therefore, a certain amount of ascorbic acid was added before cycle 2. Indeed, an enhanced hydrogen evolution was observed in the first 20 h in cycle 2, giving a similar photocatalytic activity to that in cycle 0. However, the activity slowed down afterward and an additional yellow color in the reaction solution appeared. Photocatalytic activity further decreased to 50% in cycle 3, and no further enhancement in hydrogen evolution was observed with addition of ascorbic acid in cycle 4. Considering the stable reaction solution that we had, we assume that instead of photodegradation of the ternary Pdots, the decrease in photocatalytic activity is most likely due to the colored oxidized ascorbic acid (sacrificial donor) with high extinction coefficient,^58^ competing for photon absorption with the ternary Pdots over the reaction time. To confirm our hypothesis, after the fourth cycle of photocatalytic reaction, we purified the colloidal Pdots with water by using a centrifuge tube that contains a membrane size of MWCO 15 kDa (details shown in the Supporting Information). The Pdot solution that remained in the upper part of the centrifuge tube (Figure S19, dark blue solution) was collected, and DLS and UV–vis analysis were used to study the stability of ternary Pdots. As shown in Figure 7b (dashed line), besides a minor decrease in the absorption of the ITIC peak, the UV–vis absorption of Pdots (Figure 7b, solid line) after purification gave an identical spectrum to that of the as-prepared Pdots, indicating that there is no significant degradation during photocatalysis after 120 h. The washed solution at the bottom of centrifuge tube was collected and measured with UV–vis, giving the same feature as oxidized ascorbic acid as shown in Figure S19, confirming our assumption that the colored oxidized or decomposed ascorbic acid indeed is a light absorber competing with light absorption with organic catalysts and resulting in a decayed hydrogen evolution rate over time. This result implies that in addition to achieving a highly efficient photocatalytic system for hydrogen evolution, a design for a full water splitting system is urgently needed in order to avoid using sacrificial donors. Pdots remained highly stable even after several times of centrifugation. As shown in Figure 7c, DLS measurement gives a similar size to as-prepared Pdots. The photocatalytic activity of this purified Pdot solution was further checked by adding ascorbic acid and purging with Ar. The corresponding photocatalytic data are collected in Figure 7d; the purified Pdots retained a high photocatalytic activity. Cryo-TEM analysis also indicates that Pt NPs are well absorbed by Pdots after photocatalysis reactions and purification steps (Figure S20) without any aggregation. After another 24 h of photocatalytic reaction, the average mass-normalized HER rate of this purified sample remained 80% if we compare that with reaction cycle 0. A small decrease is probably due to some loss of Pt NPs during centrifugation, since Pt NPs are only physiosorbed on the surface of Pdots; however, this is a common limitation for colloidal nanocatalysts.^59,60^

## Conclusion

Panchromatic ternary polymer dots (Pdots) involving both energy transfer and charge transfer processes have been prepared based on three light-absorbing components, PFBT, PFODTBT, and ITIC, and successfully applied for photocatalytic hydrogen production. The morphology of the Pdots obtained from cryo-TEM suggests that the ITIC has a layered crystalline structure, which is beneficial for the interaction with the catalyst and the photocatalytic reaction. Transient absorption spectroscopy measurements show that excitation energy transfer occurred from excited PFBT to PFODTBT and ITIC within 600 fs, and charge transfer including electron and hole transfer between PFODTBT and ITIC happened within 200 fs. Thanks to the ideal morphology and efficient energy and charge transfer, the optimized system has shown an external quantum efficiency up to 7% at 600 nm, making the ternary Pdots one of the most efficient organic photocatalytic systems for hydrogen evolution. Most importantly, no obvious photodegradation of the system has been monitored after photocatalytic reaction of more than 120 h. The outstanding stability implies that the ternary Pdots may meet the requirements for green hydrogen production on a large scale, and further design of photocatalysts for overall water splitting may be urgently needed in order to avoid using sacrificial donors. The reported system could also be applicable for other photocatalytic reactions, such as CO_2_ reduction and photoredox catalysis for chemical synthesis.

## Supporting Information Available

DLS of Pdots; cryo-TEM analysis of Pdots, hydrogen evolution data point versus time of Pdots; transient spectroscopy data, spectroelectrochemistry study of polymers and acceptor ITIC; steady-state UV–vis and fluorescence spectra of Pdots and ITIC (PDF)
