# Supplementary material for: Panchromatic Ternary Polymer Dots Involving Sub-Picosecond Energy and Charge Transfer for Efficient and Stable Photocatalytic Hydrogen Evolution
Source: J Am Chem Soc. 2021 Feb 4;143(7):2875–85. doi: 10.1021/jacs.0c12654 (PMC7917435; doi:10.1021/jacs.0c12654)
Supplement: Supplementary file 1 — ja0c12654_si_001.pdf [file ja0c12654_si_001.pdf]

# Supporting information

## Panchromatic Ternary Polymer Dots Involving Sub-Picosecond Energy and Charge Transfer for Efficient and Stable Photocatalytic Hydrogen Evolution

Aijie Liu,[a] Lars Gedda,[a] Martin Axelsson,[a] Mariia Pavliuk,[a] Katarina Edwards,[a] Leif Hammarström,[a] Haining Tian [a]*

[a]Department of Chemistry-Ångström Lab., Box 523, SE 751 20, Uppsala University, Sweden

*Corresponding authors:

Haining Tian: haining.tian@kemi.uu.se

**UV-Vis absorption of all components of Pdots**

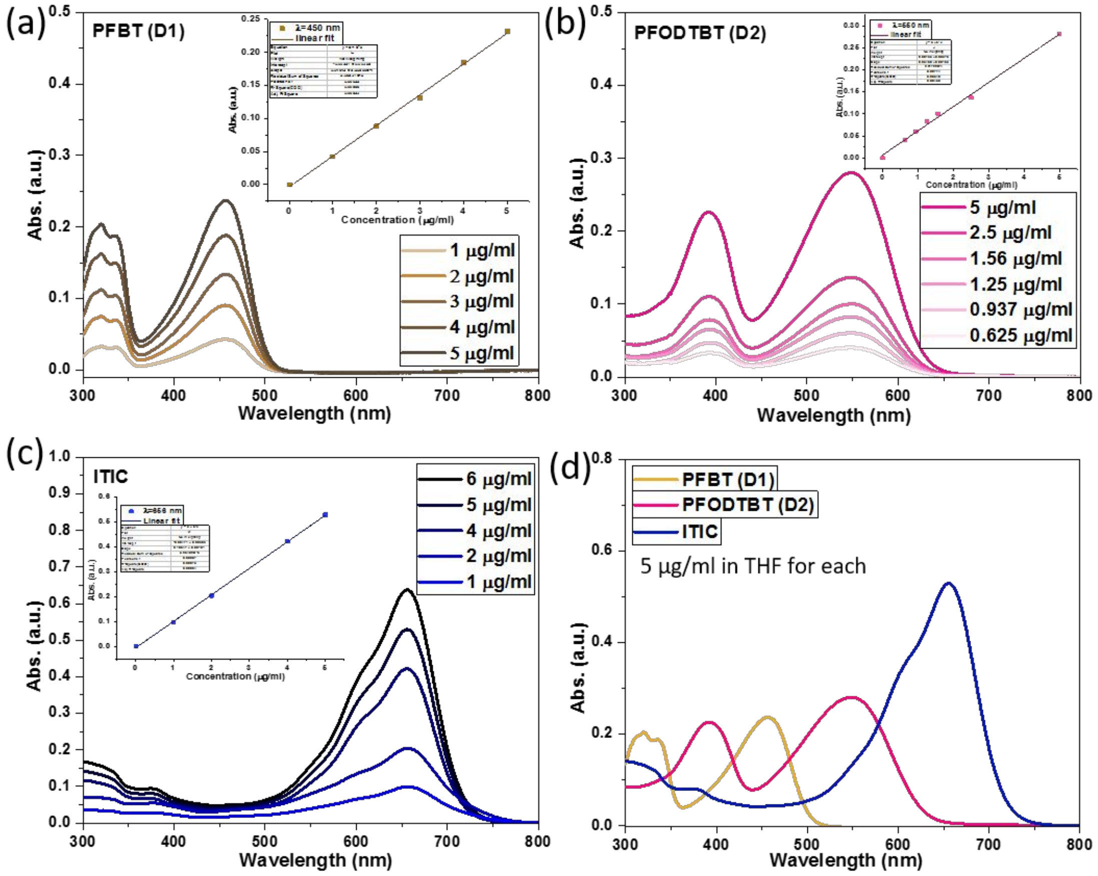


**Figure S1.** UV-Vis absorption of PFBT (D1), PFODTBT (D2) and ITIC with various concentrations in THF solution.

## DLS measurement of Pdots

DLS measurement is based on random thermal Brownian motion, that is modelled by the Stokes-Einstein equation:

$$D_\tau = \frac{k_B T}{6\pi\eta R_h} \qquad \text{eq S1}$$

Where $D_\tau$ is the diffusion coefficient, $k_B$ is Boltzmann coefficient ($1.38 \times 10^{-23}$ kg.m$^2$.s$^{-2}$.K$^{-1}$), T is an absolute temperature, and $\eta$ is the viscosity of the medium, $R_h$ is the hydrodynamic radius of a hypothetical sphere that diffuses at the same rate as particle under investigation.[1]

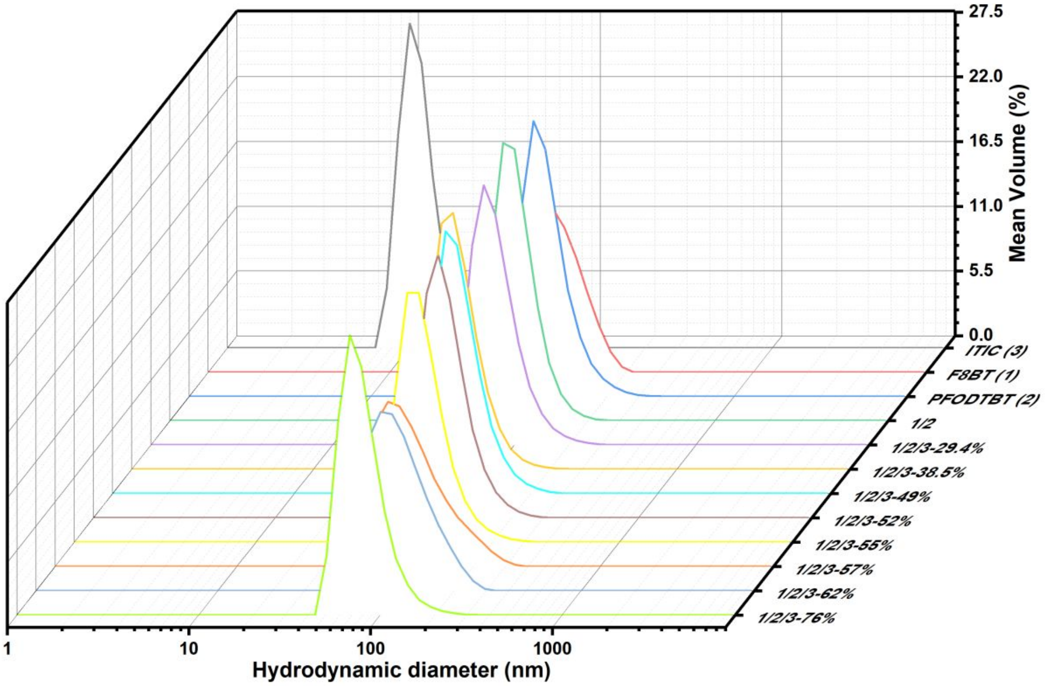


**Figure S2.** Dynamic light scattering (DLS) analysis of ITIC NPs, PFBT ($D_1$) NPs, PFODTBT ($D_2$) Pdots, $D_1/D_2$ binary Pdots and ternary Pdots with various amount of ITIC.

**Cryo-TEM analysis of Pdots**

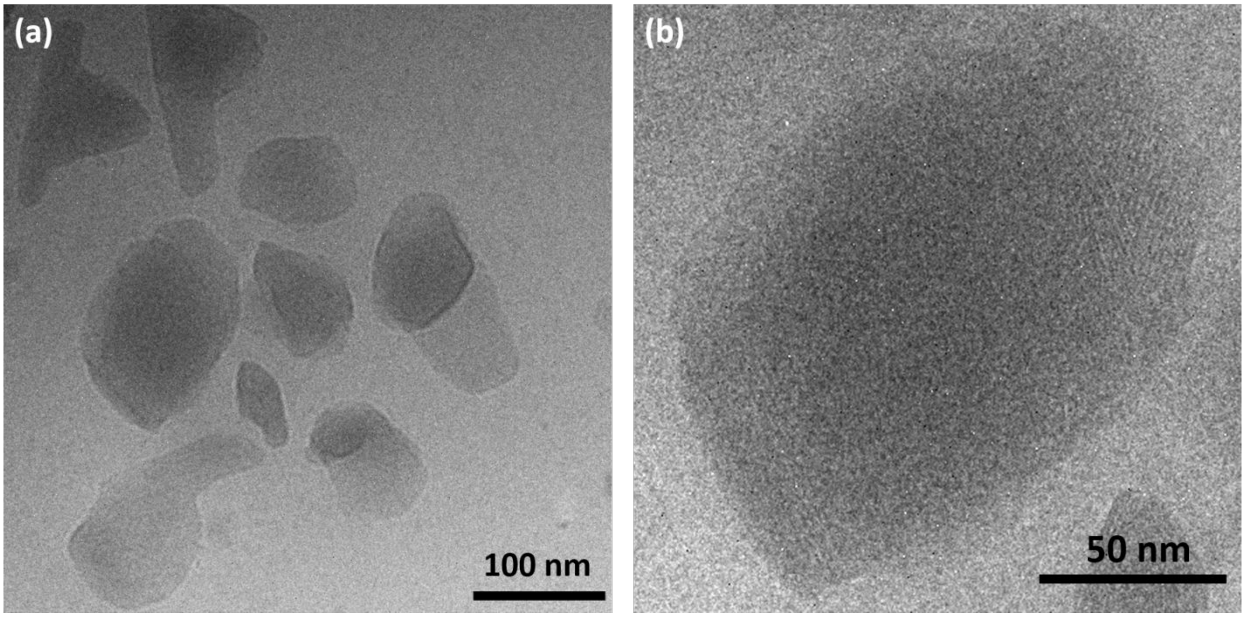


**Figure S3.** Cryo-TEM image showing (a) overview, and (b) close-up view of $D_1/D_2$/ITIC ternary Pdots.

**Powder X-ray Diffraction (PXRD)**

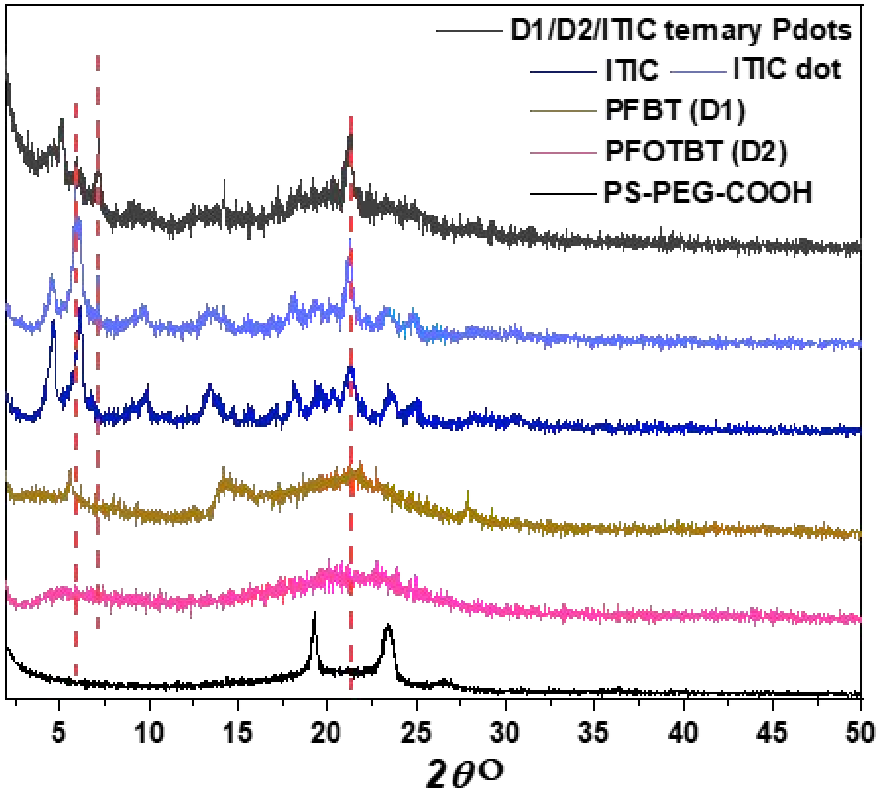


**Figure S4.** Powder X-ray diffraction (PXRD) patterns of ternary Pdots, ITIC dots, powder ITIC, PFBT ($D_1$), PFODTBT ($D_2$) and surfactant PS-PEG-COOH.

Powders of $D_1$/$D_2$/ITIC ternary Pdots, ITIC dots (PS-PEG-COOH surfactant stabilized dots), ITIC, PFBT ($D_1$), PFODTBT ($D_2$) and surfactant PS-PEG-COOH were analysed by PXRD measurements. The featureless patterns observed for both $D_1$ and $D_2$ indicate their amorphous nature. In contrast, diffraction patterns for ITIC powder and ITIC dots have sharp diffraction peaks, which is in good agreement with crystalline phase of this material. PXRD pattern of ternary Pdots maintain features from crystalline ITIC and amorphous polymers accordingly. The diffraction peaks of ternary Pdots, ITIC dots and ITIC at 2θ of 21.41° corresponding to π-π stacking of ITIC backbones; peaks at 5.94° and 7.14° corresponding to lamellar packing.

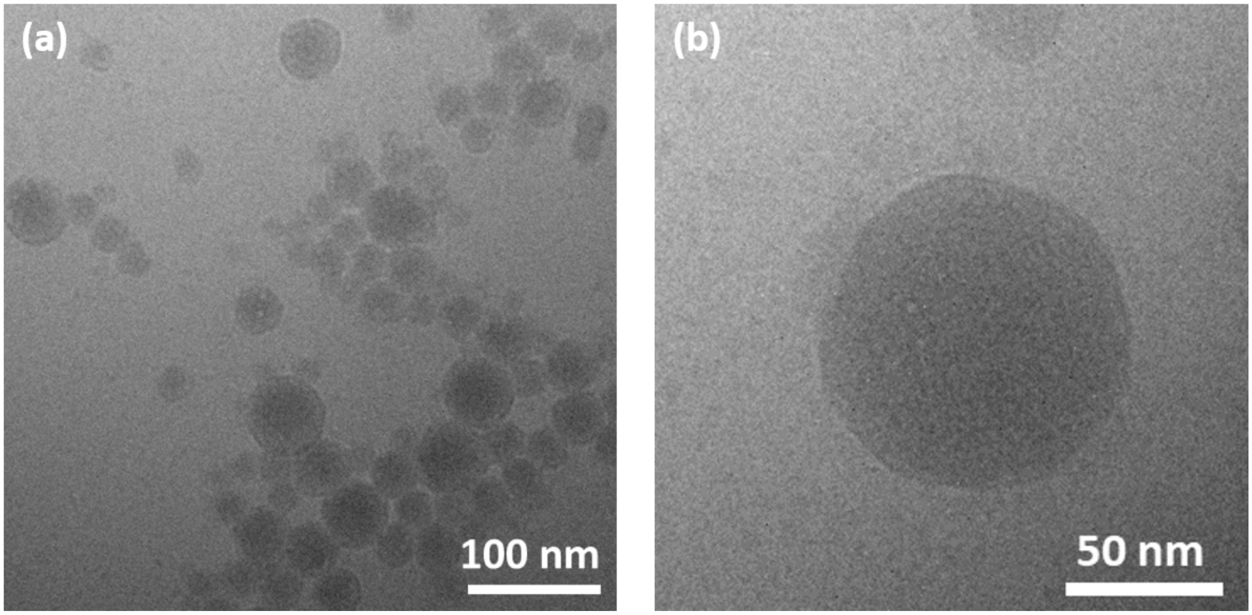


**Figure S5.** Cryo-TEM micrograph showing a) overview, and b) close-up view of $D_1/D_2$ binary Pdots. Note spherical shape and porous, amorphous structure of the Pdots.

**Hydrogen evolution:**

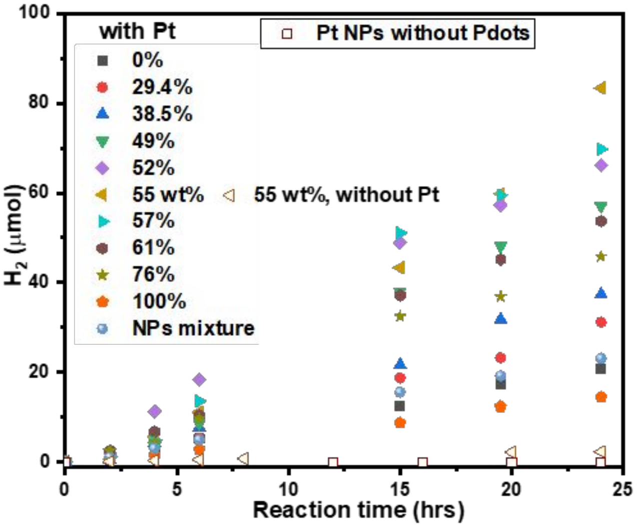


**Figure S6.** Hydrogen evolution versus time including ternary Pdots with various amount of ITIC, Reaction condition: Ternary Pdots 62 µg with 6 wt% of Pt (4 µg) in 2 mL of 0.2 M ascorbic acid solution, pH4, in 9 mL air-tight vial; ternary Pdots with 55wt% ITIC without Pt cocatalyst, in 2ml of 0.2 M ascorbic acid solution, pH4, in 9 mL air-tight vial; and $K_2PtCl_4$ (equal to 4µg Pt) in 0.2 M ascorbic acid, pH4, in 9 mL air-tight vial. Pt NPs without Pdots were performed with Pt precursor (equal to 4µg of Pt NPs) in 2ml of water solution contains 0.2 M ascorbic acid, pH4 in 9 mL air-tight vial.

## TAS study of individual Pdots

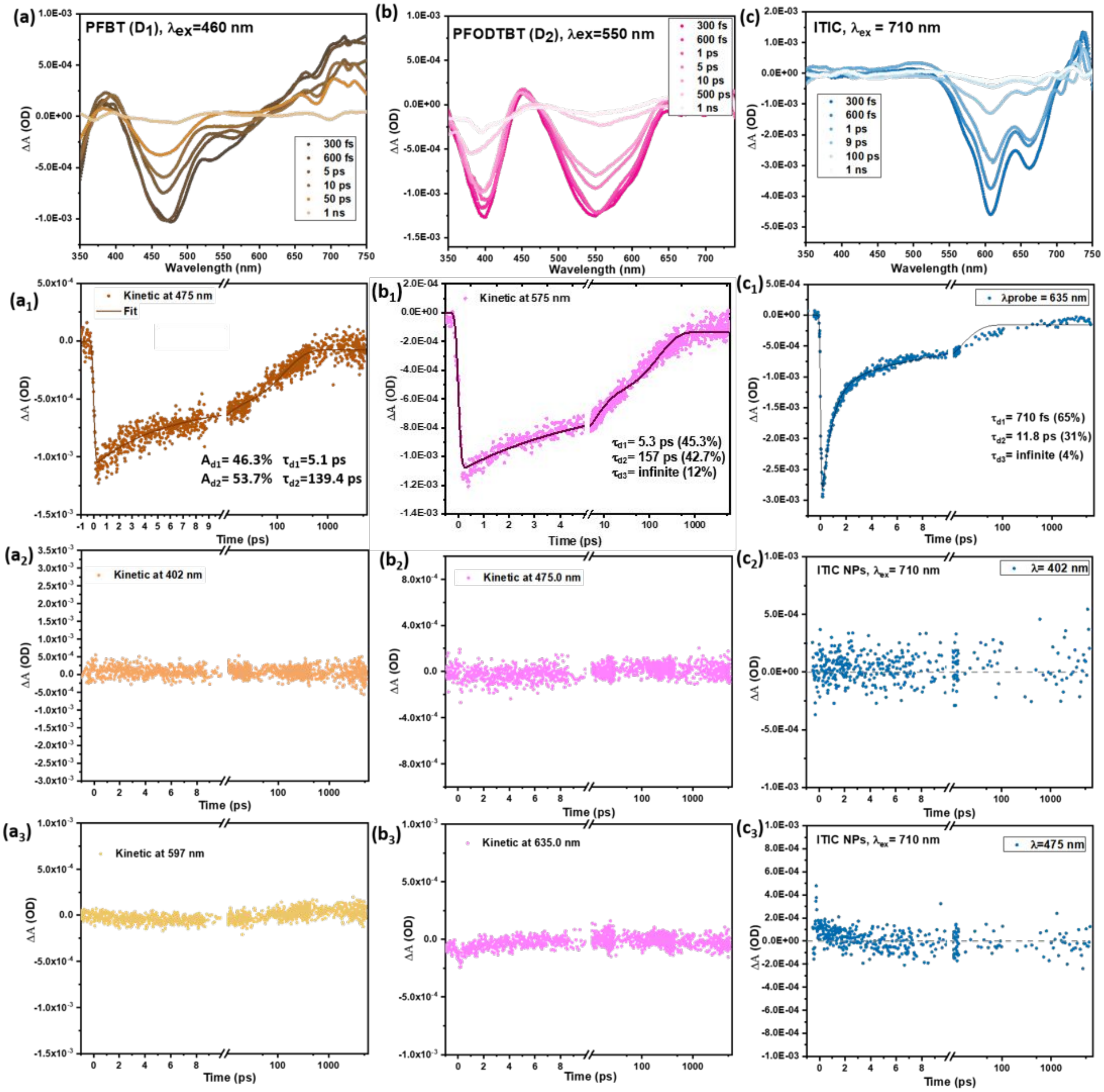


**Figure S7.** TA spectrum and their kinetics of (a) PFBT ($D_1$) Pdots under excitation of 460 nm, pump power 80 µW; (b) PFODTBT ($D_2$) Pdots, excitation 550 nm, pump power 80 µw; (c) ITIC under excitation of 710 nm, pump power 170 µw.

## Spectroelectrochemical spectroscopy

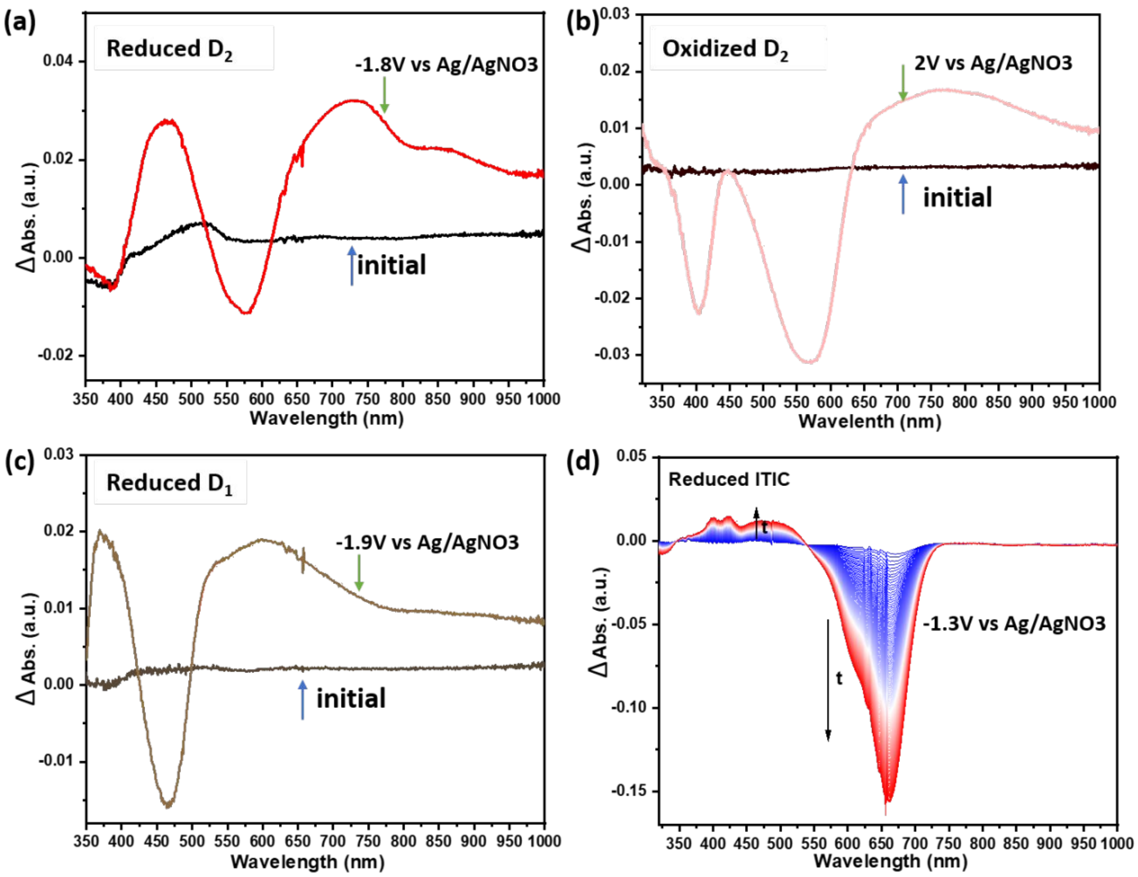


**Figure S8.** Spectroelectrochemical measurements were carried out as follow: polymers were coated on FTO glasses work as working electrode within solvent of acetonitrile, (a) reduced PFODTBT ($D_2$) with potential of -1.8 V vs $Ag/AgNO_3$ was applied; (b) oxidized $D_2$ with potential of 2 V vs $Ag/AgNO_3$ was applied; (c) reduced PFBT ($D_1$) with potential of -1.9 V vs $Ag/AgNO_3$ was applied; (d) spectrum of reduced ITIC was obtained with ITIC THF solution, potential -1.3 V vs $Ag/AgNO_3$ was applied.

## UV-vis absorption and Fluorescence spectroscopy

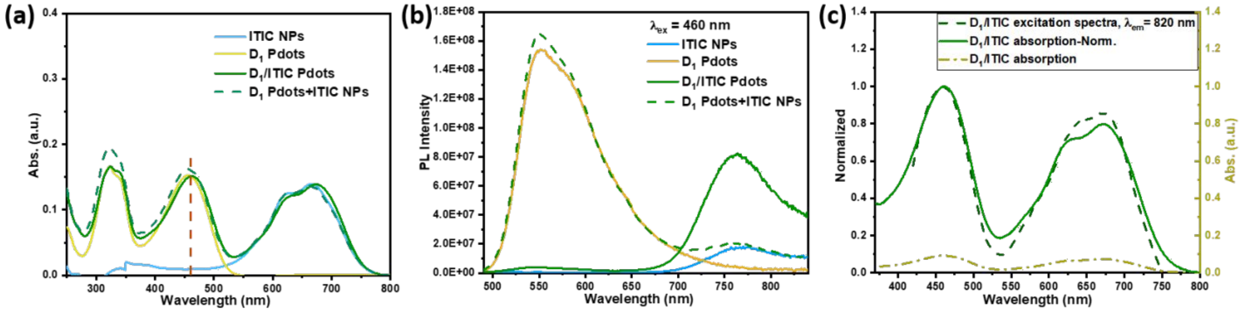


**Figure S9.** Steady-state of (a) UV-Vis absorption of ITIC NP, $D_1$ Pdots and $D_1$/ITIC binary Pdots and individual $D_1$ Pdots + ITIC NPs mixture with same concentration of binary Pdots; (b) emission spectrum of NPs with excitation at $\lambda_{ex}$=460 nm; (c) Normalized absorption, excitation spectrum ($\lambda_{em}$=820 nm).

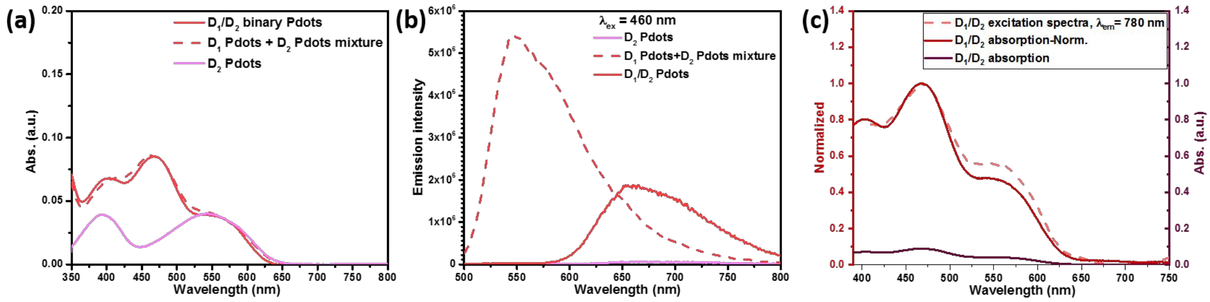


**Figure S10.** Steady-state of (a) UV-Vis absorption and (b) emission spectrum of $D_2$ Pdots, $D_1/D_2$ binary Pdots and $D_1$ Pdots+$D_2$ Pdots mixture with same ratio as $D_1/D_2$ binary Pdots. (c) Normalized absorption and excitation spectrum ($\lambda_{em}$=780 nm) of $D_1/D_2$ binary Pdots.

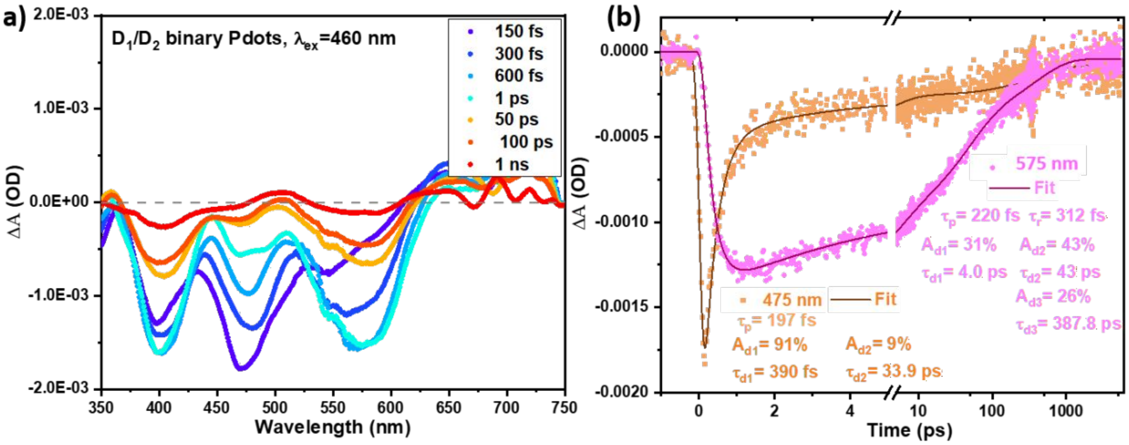


**Figure S11.** (a) Transient absorption (TA) spectra of $D_1/D_2$ binary NPs; (b) kinetic studies of (a).

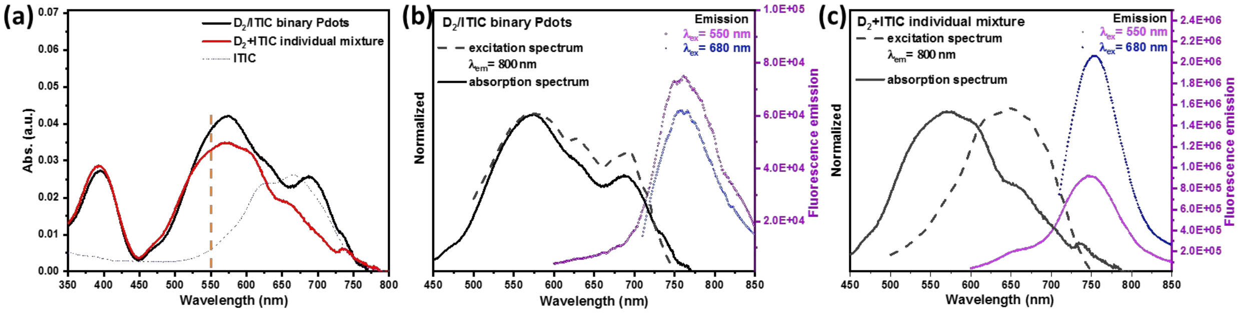


**Figure S12.** Steady-state of (a) UV-Vis absorption of ITIC NP, $D_2$/ITIC binary Pdots and $D_2$ Pdots + ITIC NPs individual mixture with same component concentration of binary Pdots; (b) Fluorescence emission spectrum of $D_2$/ITIC binary Pdots with excitation at 550 nm and 680 nm, excitation spectrum with with emission at 800 nm; (c) Fluorescence emission spectrum of $D_2$+ITIC individual mixture with excitation at 550 nm and 680 nm, excitation spectrum with emission at 800 nm.

**TAS analysis on Ternary Pdots with and without Pt NPs:**

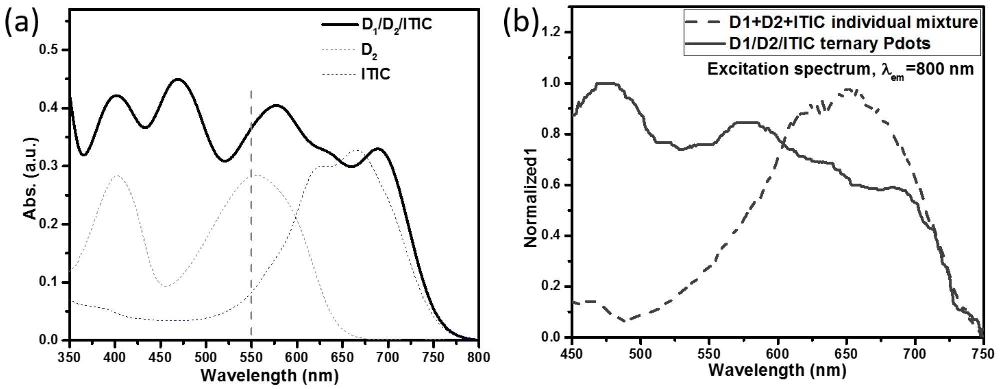


**Figure S13.** (a) UV-Vis of $D_1/D_2$/ITIC ternary Pdots with ITIC of 29 wt% (solid black line), dashed lines indicate each component. 80% of photon was absorbed by $D_1$ under excitation of 460 nm. 84% of photon was absorbed by $D_2$ under excitation of 550 nm. (b) Excitation spectrum of ternary Pdots and individual mixture at equal composition of ternary Pdots with emission wavelength at 800 nm.

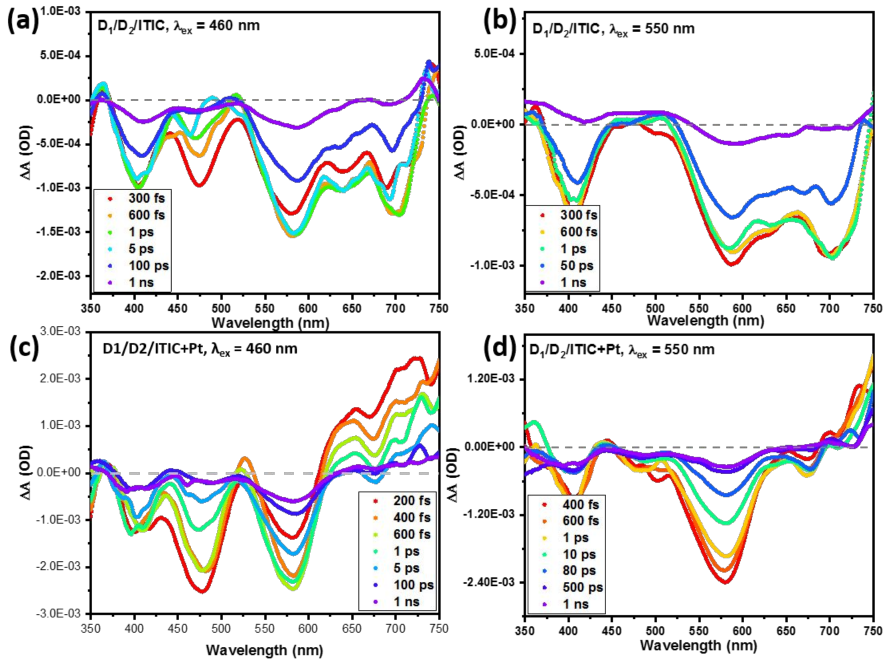


**Figure S14.** TA spectra of $D_1/D_2$/ITIC ternary Pdots with 29 wt% of ITIC under the excitation of (a) 460 nm, b) 550 nm; in-situ Pt deposited $D_1/D_2$/ITIC ternary Pdots under the excitation of (c) 460 nm, (d) 550 nm, pump power 80 μw.

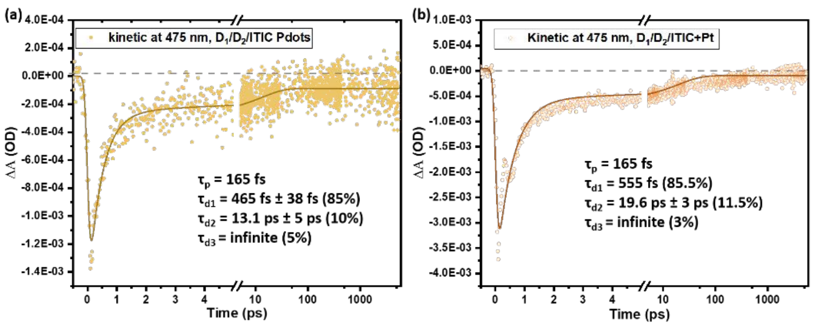


**Figure S15.** Kinetic studies and comparison of GSB of $D_1$ within $D_1/D_2$/ITIC ternary Pdots under the conditions of with and without Pt NPs, under the excitation of 460 nm.

According to absorption spectrum of ternary Pdots, shown in Figure S13a, 80% of photon was absorbed by $D_1$ under excitation of 460 nm. The kinetic traces of GSB recovering for $D_1$ are shown in Figure S15, the kinetics were fitted by using a sum of exponential functions convoluted with the instrumental response function. Within the first picosecond time range, a fast recovering of $D_1$ GSB at the peak of 475 nm was observed in systems both with and without Pt NPs, accompanying with an increase in the GSB intensity of $D_2$ (peak at 575 nm) and ITIC (625nm to 725 nm), shown in Figure S14a & c, indicate an energy transfer (EnT) process from $D_1$* to $D_2$ and ITIC. Kinetic traces of $D_1$ GSB recovering indicate that at least 85% of photon energy absorbed by $D_1$ was transferred to $D_2$ and ITIC in the time components of 600 fs or less. Similar kinetics of $D_1$ GSB recovering in systems both with and without Pt cocatalyst were found, this may suggest no direct reaction between $D_1$ and Pt within the timescale we studied.

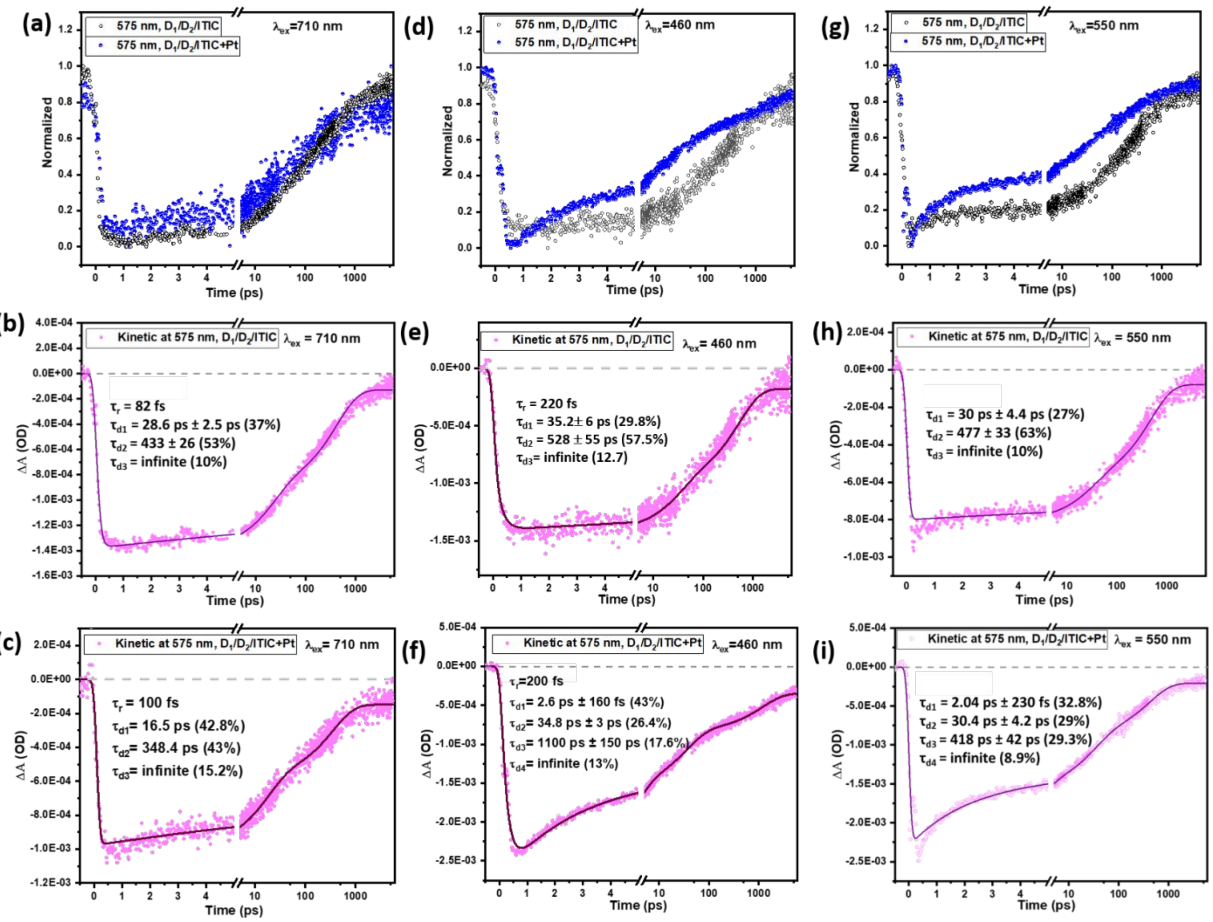

**Figure S16.** Kinetic studies of GSB of $D_2$ within $D_1/D_2$/ITIC ternary Pdots under the conditions of with and without Pt NPs the excitation of (a), (b) and (c) with 460 nm; (d), (e) and (f) with 550 nm; (g), (h) and (i) with 710 nm. (a), (d) and (g) are normalized kinetic traces. All kinetics were fitted by using a sum of exponential functions convoluted with the instrumental response function.

For the convenience of a direct comparison, we normalized the kinetics traces of $D_2$ GSB probed at 575 nm, shown in Figure S16 a, d, g, which are excited at 710 nm, 460nm and 550 nm, respectively. While the actual kinetics shown in Figure S16 b & c for system with and without Pt NPs under excitation of 710 nm, respectively; Figure S16 e & f for system with and without Pt NPs under excitation of 460 nm, respectively; and Figure S16 h & i for system with and without Pt NPs under excitation of 550 nm, respectively.

Under the excitation of 710nm, ITIC was selectively excited, according to the energy level of three components, photoinduced hole transfer (HT) from ITIC* to $D_2$ is then the only photophysical pathway in ternary system. The observation for this ternary Pdots was similar with the binary $D_2$/ITIC system, that immediately after the excitation, the GSB of $D_2$ appeared along with ITIC, with rise time ($\tau \approx 100$ fs) below our instrument response function (IRF, $\tau \approx 200$ fs), as shown in Figure S16 b & c, indicate an ultrafast hole transfer process from ITIC* to $D_2$ in the ternary Pdots. Similar $D_2$ GSB recovering kinetics were observed for systems with and without Pt NPs.

Under the excitation of 460 nm, 80% of photon is absorbed by $D_1$, therefore $D_2$ and ITIC GSB intensity enhancement is mainly a result from EnT of the excited $D_1$ ($D_1$*). Again, similar with binary Pdots, both the recovery dynamics of $D_1$ GSB and the formation dynamics of the excited ITIC and $D_2$ reflect the EnT rate. The observed rise time of $D_2$ GSB shows 200 fs in both systems with and without Pt NPs, as shown in Figure S16 e & f, respectively. Which may indicate a fine phase intermix between $D_1$ and $D_2$ in ternary Pdots.

Under the excitation of 550 nm, the kinetic traces at 575 nm indicate the recovering of excited $D_2$ and oxidized $D_2$ (a result of both electron transfer from $D_2$* to ITIC and hole transfer from ITIC* to $D_2$). A faster recombination is observed in the system with Pt (Figure S16i) compare to system without Pt (Figure S16h). The possible mechanism of this faster recombination was explained detail in the main text.

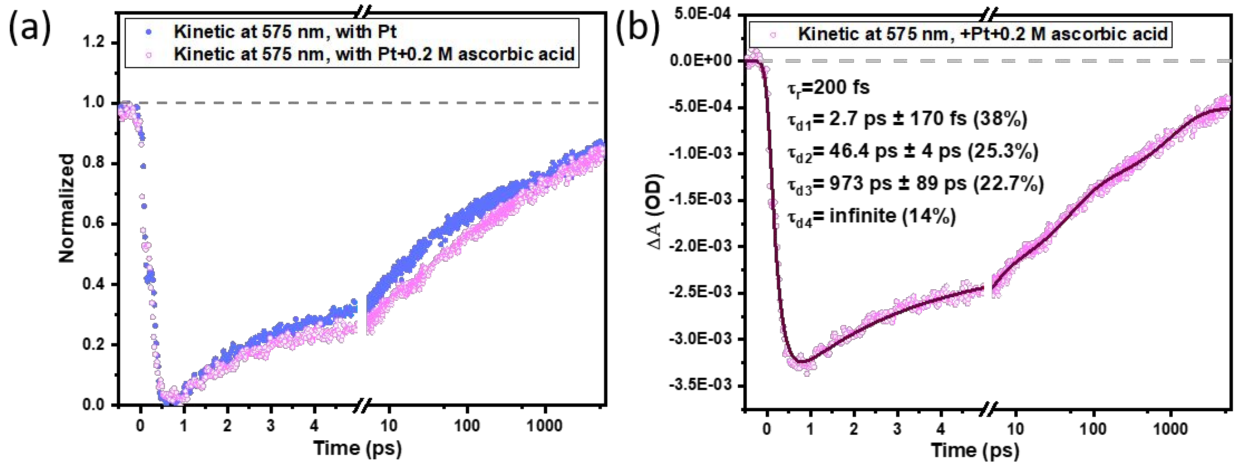


**Figure S17.** Comparison of decay kinetics of $D_2$ GSB at 575nm under the excitation of 460 nm, systems with and without ascorbic acid were compared (a) normalized kinetics for the direct comparison; (b) actual kinetics at 575 nm for ternary Pdots+Pt under 0.2 M ascorbic acid.

The kinetics traces of $D_2$ GSB was studied for the ternary Pdots with in-situ Pt deposited, under 0.2 M ascorbic acid. Under the excitation of 460 nm, kinetics trace of $D_2$ GSB was probed at 575 nm in order to compare result obtained in Figure S16f. For the convenient of a direct comparison, kinetic traces were normalized, as shown in Figure S17a. A slight slower recombination for system under ascorbic acid was observed (Figure S17a purple empty circle) compare to system without ascorbic acid (Figure S17a blue circle). For the system under ascorbic acid, the actual kinetics curve shown in Figure S17b. A slight enhancement in lifetime at picosecond timescale was observed.

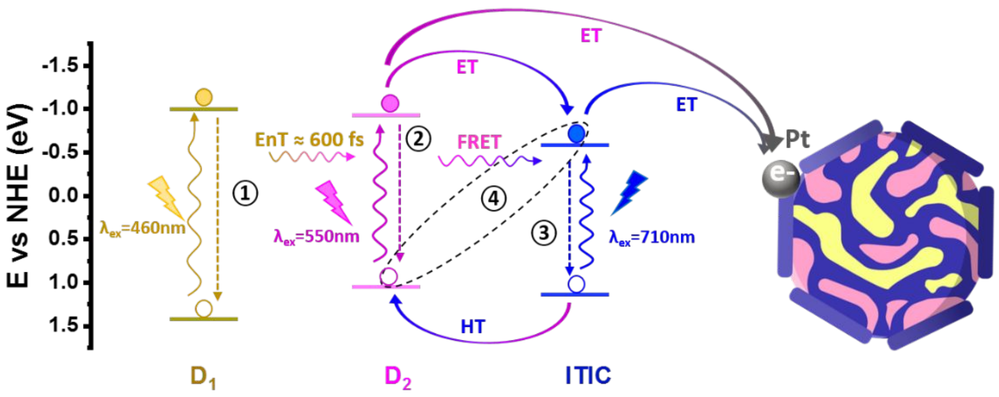


**Figure S18.** Energy and charge transfer pathways between $D_1$, $D_2$, ITIC and Pt cocatalyst.

Same photophysical pathways were found as indicated in the primary framework. Under excitation of 460 nm, EnT (include both Dexter EnT and FRET) from $D_1$* to $D_2$; secondly, one step ET from $D_2$* to ITIC; and/or two-step process involving FRET from $D_2$* to ITIC, followed with HT from ITIC* to $D_2$. In parallel, EnT from $D_1$* to ITIC and followed with HT from ITIC* to $D_2$ is also possible. With excitation of 550 nm, again, both one step of ET from $D_2$* to ITIC, and/or two-step process are possible. While under excitation of 710 nm, photoinduced HT is the only reaction that can happen from the ITIC* to $D_2$.

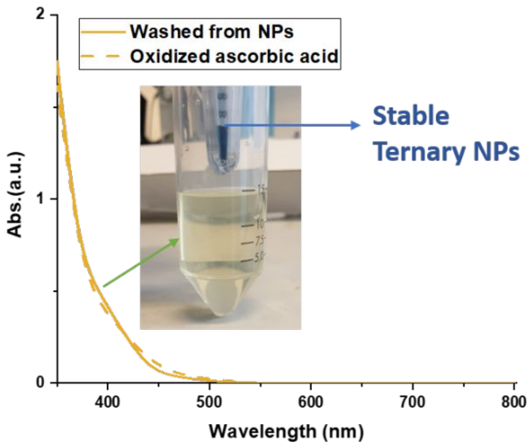


**Figure S19.** UV-Vis of solution washed from reaction solution and oxidized ascorbic acid as reference.

After the fourth cycle of photocatalytic reaction, ternary Pdots was washed with water by using centrifuge tube with membrane size of MWCO 15 kDa. Ternary Pdots was remained in the top, within the membrane. While small molecules such as ascorbic acid or oxidized ascorbic acid can be washed away and collected in the bottom of the tube. Samples were washed with 100 times of volume in total in order to remove (oxidized) ascorbic acid completely.

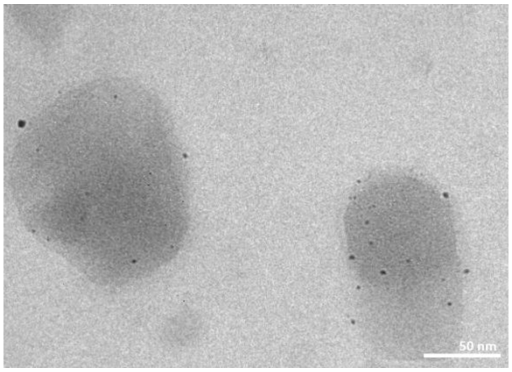


**Figure S20.** Cryo-TEM of in-situ Pt deposited ternary Pdot after purification with water by using centrifuge filter with size cut-off MWCO 15 kDa.

Reference

(1) Stetefeld, J.; McKenna, S. A.; Patel, T. R. Dynamic Light Scattering: A Practical Guide and Applications in Biomedical Sciences. *Biophys. Rev.* **2016**, *8* (4), 409–427.
